# Immune-evading universal donor iPSC-derived natural killer cells enable off-the-shelf cancer immunotherapy

**DOI:** 10.3389/fimmu.2026.1938206

**Published:** 2026-10-29

**Authors:** Masayuki Ito, Hirofumi Tsuzuki, Michael A. Secinaro, Shannon Kreuser, Kaori Oda, Borith Kheng, Louie Galitan, Julie A. Vergara, Ippei Takeuchi, Sarah L. Ameny, Mary G. Prieve, Mark T. Orr, Taku Yoshida

**Affiliations:** 1 Research, Universal Cells, Inc., Seattle, WA, United States; 2 Research, Astellas Institute for Regenerative Medicine, Westborough, MA, United States

**Keywords:** cancer, cell therapy, immune evasion, induced pluripotent stem cells, natural killer cells, universal donor cells

## Abstract

**Background:**

Natural killer (NK) cells are a promising modality for cancer immunotherapy due to their intrinsic ability to eliminate malignant cells and their safety profile, while their clinical application remains limited by donor variability, immune rejection, and challenges in large-scale manufacturing.

**Methods:**

We developed a multi–gene-edited universal donor induced pluripotent stem cell (iPSC)-derived NK cell (UDC-NK) platform. First, the engineered iPSC line was generated by disrupting B2M and RFXANK to eliminate HLA class I and II molecules and by introducing a single-chain HLA-E for immune evasion. In addition, a safety switch system was incorporated to ensure controllability. We also developed a novel feeder-free, scalable manufacturing process for cryopreserved UDC-NK cells for off-the-shelf cellular therapy.

**Results:**

We succeeded in generating large numbers of UDC-NK cells with an activated phenotype. UDC-NK cells exhibited robust cytotoxicity and cytokine production against CD20^+^ lymphoma cell lines *in vitro*, which were further enhanced in the presence of Rituximab. UDC-NK cells effectively demonstrated immune evasion from allogeneic T cell– and NK cell–mediated responses. In addition, UDC-NK cells could be selectively eliminated via a herpes simplex virus thymidine kinase–based safety switch upon ganciclovir treatment *in vitro*. In disseminated B-cell lymphoma models, combination treatment with UDC-NK cells and Rituximab resulted in near-complete tumor growth inhibition and significantly prolonged survival.

**Conclusions:**

These findings establish UDC-NK cells as a scalable off-the-shelf cell therapy platform with enhanced anti-tumor efficacy and built-in safety control, supporting their potential clinical application in combination with antibody-based cancer therapies.

## 1 Introduction

Cancer immunotherapy has rapidly advanced in recent years and is now widely recognized as a major pillar of cancer treatment alongside surgery, chemotherapy, targeted therapy, and radiotherapy. The development of immune checkpoint inhibitors and adoptive cell therapies has significantly improved outcomes for patients with several malignancies (1–3). Immune cell-based cancer therapies have also shown remarkable clinical success in hematological cancers. Autologous chimeric antigen receptor (CAR) T-cell therapies targeting CD19 and BCMA have produced durable responses in patients with relapsed or refractory B-cell and plasma cell malignancies and have transformed the treatment landscape for diseases such as acute lymphoblastic leukemia, diffuse large B-cell lymphoma, and multiple myeloma (2). These successes have established adoptive cell therapy as a powerful modality for cancer treatment and have stimulated intense interest in the development of next-generation cellular immunotherapies. Despite these advances, the broader clinical application of autologous CAR-T cell therapies remains limited by several practical and biological challenges (1, 3, 4). Manufacturing of autologous products requires individualized production from each patient, involving complex and labor-intensive processes that contribute to high treatment costs. In addition, the manufacturing timeline for autologous therapies typically requires several weeks, which can delay treatment for patients with rapidly progressing diseases. Product variability resulting from differences in patient-derived starting material can also affect therapeutic consistency and efficacy. Furthermore, autologous T-cell therapies are associated with potentially severe toxicities, including cytokine release syndrome and neurotoxicity. These limitations have stimulated growing interest in alternative immune cell platforms that enable scalable, standardized, and readily available cellular therapies (3).

Natural killer (NK) cells are innate immune lymphocytes that play a critical role in the immune surveillance against infected or transformed cells. Unlike T cells, NK cells do not require antigen-specific priming and can recognize malignant cells through a balance of activating and inhibitory receptor signals (5, 6). This mechanism enables NK cells to detect stressed or transformed cells that have downregulated major histocompatibility complex (MHC) molecules, a phenomenon commonly referred to as “missing-self” recognition (7). Upon activation, NK cells exert potent cytotoxic effects through multiple mechanisms, including the release of perforin and granzymes, as well as death receptor–mediated pathways such as Fas ligand and TRAIL. In addition to direct tumor cell killing, NK cells produce cytokines such as interferon-γ (IFN-γ) and tumor necrosis factor-α (TNF-α), which can enhance antitumor immunity and promote interactions with other immune cell populations (8). NK cells can also eliminate antibody-coated tumor cells through antibody-dependent cellular cytotoxicity (ADCC) mediated by Fc receptors, allowing them to cooperate with therapeutic monoclonal antibodies (9, 10). These diverse mechanisms make NK cells an attractive cellular platform for cancer immunotherapy. However, current NK cell–based therapies face several limitations that hinder their widespread clinical implementation. Primary NK cells derived from peripheral blood or cord blood often exhibit donor-to-donor variability and limited expansion potential, complicating large-scale manufacturing. In addition, NK cells typically show limited persistence after infusion, which may restrict long-term therapeutic efficacy. NK cells are also sensitive to cryopreservation, which can impair their biological activity and antitumor efficacy post-thaw. Consequently, generating consistent, scalable, cryopreserved NK cell products suitable for clinical use remains a major challenge for the field (11–13).

Induced pluripotent stem cells (iPSCs) offer a promising strategy to address many of these limitations. iPSCs possess the capacity for unlimited self-renewal and can differentiate into a wide range of cell types, including NK cells (14, 15). Importantly, iPSC-based platforms enable the generation of large numbers of standardized cellular products derived from a well-characterized master cell bank, thereby facilitating scalable manufacturing of off-the-shelf cell therapies (15). In addition, iPSCs provide an ideal platform for extensive genetic engineering at the pluripotent stage, allowing the introduction of multiple genetic modifications designed to enhance the efficacy, persistence, and safety of derived immune cells. These advantages make iPSC-derived immune cells an attractive approach for developing next-generation cellular immunotherapies (16–18). More recently, universal donor iPSC (UDC-iPSC) technologies using adeno-associated virus (AAV)–mediated gene editing have been developed to overcome immune rejection following allogeneic cell transplantation. By disrupting the B2M and RFXANK genes, which are required for surface expression of HLA class I and class II molecules, respectively, and introducing a single-chain HLA-E construct, UDCs and their differentiated derivatives can evade recognition by host T cells while avoiding NK cell–mediated rejection (19–21). These strategies have the potential to generate broadly compatible allogeneic cellular therapeutics that can be administered across multiple patients without HLA matching. Consequently, UDC technologies represent a promising approach for enabling universal, off-the-shelf cell therapy platforms.

Recent advances in iPSC-derived NK cell technologies have further accelerated the development of next-generation, off-the-shelf cellular immunotherapies (16–18). Several groups have demonstrated the feasibility of generating large numbers of functional NK cells from iPSCs under clinically scalable manufacturing conditions, leading to the emergence of multiple clinical-stage iPSC-NK cell products (15, 17, 22, 23). In parallel, extensive genetic engineering strategies have been implemented to enhance the therapeutic performance of iPSC-derived NK cells. These approaches include the introduction of high-affinity and non-cleavable CD16 variants to improve antibody-dependent cellular cytotoxicity (ADCC) (17, 21), expression of cytokine support systems such as membrane-bound IL-15 to enhance persistence and functionality (17, 22, 23), incorporation of chimeric antigen receptors (CARs) to increase tumor specificity (16, 17), and genetic modifications designed to improve resistance to the immunosuppressive tumor microenvironment (22, 23). More recently, immune-evasive engineering strategies, including modulation of HLA molecules and expression of inhibitory ligands, have been developed to reduce host immune rejection and prolong persistence following allogeneic administration (19, 23–25). Collectively, these advances have established genetically engineered iPSC-derived NK cells as a highly promising platform for the development of universal, off-the-shelf cancer immunotherapies capable of combining enhanced persistence, immune evasion, and potent antitumor activity.

In this study, we developed universal donor iPSC (UDC-iPSC)-derived NK cells (UDC-NK) designed to enhance immune evasion, safety, and antitumor efficacy. The engineered UDC clone incorporates multiple genetic modifications, including disruption of *B2M* and *RFXANK* to eliminate HLA class I and class II expression, expression of a single-chain HLA-E construct to prevent NK cell–mediated rejection, introduction of a high-affinity *FCGR3A* (F158V) variant to enhance ADCC (21), and integration of a herpes simplex virus thymidine kinase (HSV-TK) suicide gene as an inducible safety switch (26–28). We also established a feeder-free and scalable manufacturing process capable of generating large numbers of functional NK cells. Using this platform, we efficiently generated NK cells from UDCs and comprehensively characterized their phenotype and function *in vitro* and *in vivo*. We evaluated surface marker expressions, cytotoxic effector functions including natural cytotoxicity and ADCC, cytokine production, and the immune-evasive properties of the engineered NK cells in the context of allogeneic immune recognition. In addition, we validated the functionality of the integrated HSV-TK suicide gene as an inducible safety switch *in vitro*. Finally, the antitumor activity of UDC-NK cells was assessed *in vitro* and validated *in vivo* using xenograft tumor models to determine their therapeutic potential. Collectively, this study establishes a genetically engineered iPSC-derived NK cell platform that may enable the development of broadly compatible, off-the-shelf NK cell therapies for cancer.

## 2 Materials and methods

### 2.1 Cells and cell culture

The human WT iPSC line NIH3 c.C4 was established by single-cell cloning in-house from a clinical-grade iPSC bank (Lonza, Basel, Switzerland) and used for gene editing. All parental and gene-edited iPSC lines were maintained on Vitronectin XF™ (Stemcell Technologies, Vancouver, BC, Canada)–coated culture vessels in mTeSR1 or TeSR-E8 medium (Stemcell Technologies) in a 37 °C, 5% CO_2_, and 5% O_2_ incubator. Chronic myelogenous leukemia cell line K562, CD20^+^ non-Hodgkin lymphoma (NHL) cell lines Raji, Ramos, Daudi, Mino, SU-DHL-4, SU-DHL-5, SU-DHL-6, P3HR-1 cells were obtained from American Type Culture Collection (Manassas, VA, USA). Granta-519 NHL cell line was purchased from Creative Bioarray (Shirley, NY, USA). K562 cells engineered to express HiBiT protein were purchased from Promega (Madison, WI, USA). All cells were cultured in RPMI 1640 media supplemented with 2 mM GlutaMAX™ (Thermo Fisher Scientific, Waltham, MA, USA) and with 10% heat-inactivated fetal bovine serum (FBS, Thermo Fisher Scientific). For ADCC assays, NucLight™ Red-expressing Ramos cells were established by transducing cells with the IncuCyte^®^ NucLight™ Red Lentivirus Reagent (Sartorius, Ann Arbor, MI, USA), followed by puromycin selection. For *in vivo* studies, Raji-Luc and Daudi-Luc cells were established by infection with IVISbrite™ Red F-luc-Puromycin Lentiviral Particles (Revvity, Waltham, MA, USA) to introduce a Red-Shifted luciferase and a GFP reporter gene, followed by single-cell cloning.

### 2.2 Gene editing to establish universal donor iPSC (UDC-iPSC)

Establishing universal donor iPSC (UDC-iPSC) engineered to express high affinity CD16A F158V variant for the manufacturing UDC-NK cells for clinical applications, has been described previously (29). Briefly, the NIH3 c.7C2 iPSC line, which was used for UDC-NK manufacturing, was generated from NIH3 c.C4 WT iPSC clone through the multiple rounds of gene-editing, which includes (1) knocking out the *B2M* and *RFXANK* genes to eliminate surface expression of HLA Class I and Class II molecules to escape allogeneic rejection mediated by T cells, (2) introduction of a single-chain HLA-E chimeric receptor to protect differentiated cells from allogeneic rejection mediated by CD94/NKG2A-expressing allogeneic NK cells (19), (3) ganciclovir-inducible thymidine kinase gene cassettes as a safety switch to reduce the risk of severe, unwanted adverse events following administration of NK cell product to cancer patients (26, 28), and (4) *FCGR3A* F158V gene coding naturally occurred high-affinity CD16A variant to improve ADCC in combination with therapeutic antibodies. iPSC line NIH2 c.2B9 was established by disrupting *B2M* genes. All gene-editing was performed using recombinant adeno-associated viruses.

### 2.3 Cell differentiation

NK cell differentiation from iPSCs was performed using a modified protocol described previously (15). Briefly, 1,000–2,000 Trypsin-adapted single iPSCs were seeded into each well of a 24-well AggreWell™ 800 plate (Stemcell Technologies) in APEL2 medium (Stemcell Technologies) supplemented with 40 ng/mL human stem cell factor (SCF; PeproTech, Rocky Hill, NJ, USA), 20 ng/mL human vascular endothelial growth factor (VEGF; R&D Systems, Minneapolis, MN, USA), and 20 ng/mL human bone morphogenetic protein 4 (BMP-4; R&D Systems) to promote embryoid body (EB) formation. Cells were cultured at 37 °C under 5% CO_2_ and 5% O_2_. Half-medium changes were performed every other day. EBs were maintained under these conditions for 14 days to promote the generation of CD34^+^ hematopoietic progenitor cells (HPCs). On day 14, HPCs were harvested in DMEM/F-12 medium (Thermo Fisher Scientific) supplemented with 15% heat-inactivated human AB serum (BioIVT, Westbury, NY, USA), 5 ng/mL IL-3 (first week only), 10 ng/mL IL-15, 20 ng/mL IL-7, 20 ng/mL SCF, and 10 ng/mL Flt3 ligand (PeproTech). In addition, 35 nM UM171 (Xcess Biosciences, San Diego, CA, USA), a pyrimidoindole-derived small molecule previously reported to promote the self-renewal and expansion of hematopoietic stem cells and progenitor cells, was added to stabilize HPCs and enhance NK cell differentiation efficiency (30–32). The HPCs were subsequently replated onto culture vessels coated with RetroNectin™ (Takara Bio, Shiga, Japan) and human Delta-like ligand 4–Fc protein (DLL4; ACRO Biosystems, Beijing, China) to facilitate Notch signaling–mediated NK lineage commitment. Cells were further cultured for 3 weeks to promote differentiation into immature CD56^+^ UDC-NK cells. Culture medium was refreshed every 3–4 days throughout the differentiation period. Cells were maintained at 37 °C under 5% CO_2_ in air during this stage. For the assessment of immune evasion in UDC-NK cells, the *B2M* KO iPSC clone NIH2 c.2B9 was differentiated into CD14^+^ myeloid cells using the STEMdiff™ Hematopoietic Kit (STEMCELL Technologies) following previously published protocols (33).

### 2.4 Activation and expansion of UDC-NK cells

CD56^+^ immature UDC-NK cells were harvested and resuspended at a density of 2x10^5^ cells/mL in CTS™ NK-Xpander™ Medium (Thermo Fisher Scientific) supplemented with 7.5% heat-inactivated human AB serum, 10 ng/mL IL-15, 10 ng/mL IL-18 (R&D Systems), and 10 ng/mL IL-21 (Peprotech). NK cells were seeded into culture vessels pre-coated with 0.89 μg/mL anti-human NKp30 antibody (BioLegend, San Diego, CA, USA), 0.89 μg/mL anti-DNAM-1 antibody (Bio-Rad Laboratories, Hercules, CA, USA), and 0.89 μg/mL RetroNectin™. Cells were cultured for 3 days at 37 °C under 5% CO_2_ in air to stimulate the maturation and proliferation of immature NK cells. After 3 days of activation with anti-NKp30 and anti-DNAM-1 antibodies, NK cells were harvested and resuspended in CTS™ NK-Xpander™ Medium containing 7.5% heat-inactivated human AB serum, 10 ng/mL IL-15, and 10 ng/mL IL-18. The cells were then transferred at a density of 4x10^5^ cells/mL to disposable spinner flasks (Corning, Corning, NY, USA) placed on a magnetic stirrer (Chemglass Life Science, Vineland, NJ, USA) for further expansion at 45–55 rpm in a 37 °C incubator with 5% CO_2_. Following initiation of spinner flask culture, an equal volume of fresh medium containing the same supplements was added to the culture. NK cells were expanded in spinner flasks for 4 days. 3-day NK Activation and 4-day NK Expansion cycle was repeated three times. After three cycles of activation and expansion, NK cells were harvested and cryopreserved in CryoStor^®^ CS-10 (Stemcell Technologies) for further characterization. At harvest, NK cell purity was typically >99% among CD45^+^CD56^+^ cells. In selected experiments, the cycle was repeated up to seven times to evaluate the robustness and scalability of the manufacturing process.

### 2.5 Flow cytometry

Flow cytometry was performed using an Attune NxT flow cytometer (Thermo Fisher Scientific), and the data were analyzed using FlowJo™ software (FlowJo, Ashland, OR, USA). Details of antibodies used in this study are provided in **Supplementary Table 1**.

### 2.6 Ganciclovir sensitivity of UDC-NK cells

To assess the function of the HSV-TK-based safety switch *in vitro*, NK cells were seeded into a 96-well flat-bottom plate in the presence of 10 ng/mL IL-15 and a titrated concentration of ganciclovir (MilliporeSigma, Burlington, MA, USA) and cultured at 37 °C in a humidified incubator with 5% CO_2_. Four days after the initiation of ganciclovir treatment, NK cell viability was measured using CellTiter^®^-Glo 2.0 Cell Viability Assay (Promega).

### 2.7 Immune evasion of UDC-NK cells from allogeneic PBMC T cells and NK cells

To assess the activation of allogeneic T cells after co-culture with UDC-NK cells, CD4^+^ and CD8^+^ T cells isolated using magnetic-activated cell sorting (Miltenyi Biotec, Bergisch Gladbach, Germany) from the peripheral blood of healthy volunteers (Bioworks NW, Seattle, WA, USA; Charles River Laboratories, Wilmington, MA, USA) were co-cultured with UDC-NK cells in 96-well plates for 3 days. The purity of T cells and NK cells was confirmed by flow cytometry prior to co-culture. After co-culture, T cell proliferation was measured using CellTiter^®^-Glo 2.0 Cell Viability Assay (Promega). To evaluate the susceptibility of UDC-NK cells to lysis by allogeneic PBMC-derived NK cells, a DELFIA^®^ Cytotoxicity Assay (Revvity) was performed. UDC-NK cells were labeled with BATDA reagent and used as target cells. BATDA-labeled UDC-NK cells were co-cultured with PBMC-derived allogeneic NK cells at titrated effector-to-target (E:T) ratios in 96-well round-bottom plates for 2 hours at 37 °C. After co-culture, supernatants were harvested, and europium release from lysed UDC-NK cells was measured using time-resolved fluorescence with a Varioskan™ LUX Plate Reader (Thermo Fisher Scientific). Experiments were performed with 3 independent biological triplicates. Specific lysis was calculated as [(experimental release − spontaneous release)/(maximum release − spontaneous release)] × 100.

### 2.8 Cytotoxicity assays

The natural cytotoxicity of NK cells was evaluated by co-culturing NK cells with K562-HiBiT cells (Promega, Madison, WI, USA) at various effector-to-target (E:T) ratios in 96-well round-bottom plates. After 4 hours of incubation, HiBiT-tagged proteins released from lysed target cells were detected using Nano-Glo^®^ HiBiT Extracellular Detection System (Promega) according to the manufacturer’s instructions. The luminescent signal was measured using a plate reader. Experiments were performed with 3 independent biological triplicates. The percentage of specific lysis is calculated as follows: %Specific lysis = (Bioluminescence in the test samples − Background signal in the absence of effector cells)/(Bioluminescence in the presence of digitonin − Background signal in the absence of effector cells) × 100. To assess antibody-dependent cellular cytotoxicity (ADCC), NK cells were co-cultured with CD20^+^ NHL cell lines engineered to express NucLight™ Red (Sartorius) at titrated E:T ratios in the presence or absence of 1 µg/mL Rituximab biosimilar (Invivogen, San Diego, CA, USA) in 96-well flat-bottom plates. NucLight Red^+^ target cells were monitored for 24 hours using an IncuCyte^®^ S3 Live-Cell Analysis System (Sartorius).

### 2.9 Cytokine secretion

To assess cytokine secretion upon tumor cell stimulation, NK cells were co-cultured with K562 or Raji cells at an E:T ratio of 1:1 in 96-well flat-bottom plates, in the presence or absence of Rituximab (1 µg/mL). After 24 hours of incubation in a 37 °C with 5% CO_2_ incubator, the culture supernatants were harvested, and IFN-γ and TNF-α concentrations were quantified using AlphaLISA^®^ Cytokine Assays (Revvity) according to the manufacturer’s instructions.

### 2.10 Murine xenograft tumor models

NOGF-IL15 (NOD.Cg-Prkdc<scid> Il2rg<tm1Sug> Fcer1g<tm1Rav> Fcgr2b<tm1Ttk> Tg(CMV-IL15)/Jic) mice, which lacks the surface expression of all Fc receptors and expresses human IL-15 under the control of the CMV promoter (32), was provided by Taconic Biosciences (Rensselaer, NY, USA) and maintained in-house under specific pathogen-free conditions. Mice were transplanted with 2x10^4^ Raji-Luc cells or 1x10^5^ Daudi-Luc cells via intravenous (i.v.) injection on day 0. The combination therapy with cryopreserved UDC-NK cells and Rituximab was initiated the next day and administered as indicated in figure legends. Cryopreserved UDC-NK cells were thawed and immediately used for treatment. Tumor burden was monitored by bioluminescence imaging (BLI) using an IVIS Lumina III Imaging System (Revvity) under 2–4% inhaled isoflurane anesthesia. Mice were carefully monitored and euthanized upon reaching humane endpoints, defined as a body weight loss of ≥20%. At the study endpoint, mice were humanely euthanized, either by cervical dislocation under deep anesthesia induced with 2–4% inhaled isoflurane or by gradual exposure to CO_2_ at a displacement rate of 30–70% of the cage or chamber volume per minute. All animal experiments were approved by the Institutional Animal Care and Use Committee of Omeros Corporation (Seattle, WA, USA) and conducted in an Association for Assessment and Accreditation of Laboratory Animal Care International (AAALAC International)-accredited facility.

### 2.11 Statistics

Dunnett’s multiple comparisons test was used for statistical analysis. Survival was analyzed using the log-rank test. All statistical analyses were performed using GraphPad Prism software (GraphPad Software, San Diego, CA, USA). P value < 0.05 was considered statistically significant, and significance was indicated by asterisks.

## 3 Results

### 3.1 Establishing a universal donor iPSC (UDC) line with a safety switch and stable high-affinity FcR expression for NK cell manufacturing

We aimed to establish a robust platform for the manufacturing of NK cells from multiple gene-edited universal donor iPSC lines to support clinical applications of universal donor cell-derived NK (UDC-NK) therapies (**Figure 1A**). To this end, we first generated universal donor iPSCs (UDC-iPSCs) engineered to eliminate the surface expression of classical HLA class I and class II molecules and to introduce single-chain HLA-E molecules using AAV-based gene editing technologies (34). The UDC technology enables UDC-derived cell therapy products to escape from allogeneic rejection mediated by T cells and CD94/NKG2A^+^ NK cells after administration to patients, as previously described (19). Using the UDC-iPSC line as the parental iPSCs, we performed additional gene editing to introduce a ganciclovir-inducible thymidine kinase safety switch, enabling elimination of UDC-derived cell therapy products in the event of severe adverse events following administration. Finally, to enhance antibody-dependent cellular cytotoxicity (ADCC), we introduced a high-affinity natural variant of the FCGR3A gene encoding CD16A, generating the iPSC line NIH3 c.7C2, which was used for NK cell manufacturing (**Figure 1B**). A GMP bank of NIH3 c.7C2 iPSCs uniformly expressed pluripotency markers OCT4, SOX2, and SSEA-3/4, indicating maintaining pluripotency, as confirmed by flow cytometry (**Figure 1C**).

**Figure 1:**
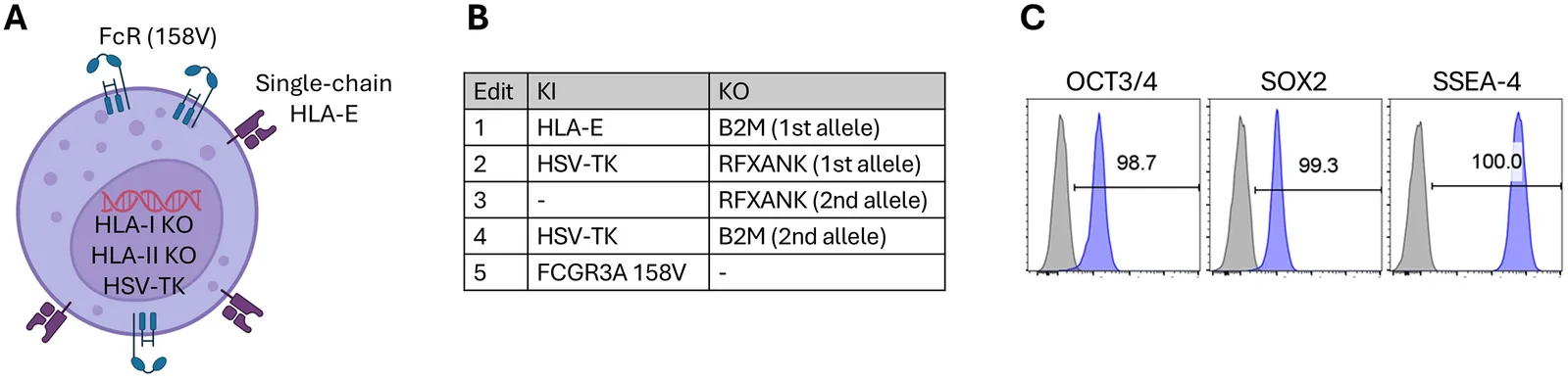
Gene editing to establish universal donor iPSCs (UDCs). **(A)** Illustration of UDC-NK cells, which lack surface expression of HLA class I and class II molecules, express HLA-E, HSV-TK safety switch, and a highly-affinity variant of CD16A. **(B)** Gene editing cascade to establish UDC-iPSC clone NIH3 c.7C2 from WT iPSC clone. **(C)** Marker phenotype of UDC-iPSC NIH3 c.7C2 (blue) compared to isotype control (grey) by flow cytometry.

### 3.2 A Highly-efficient, scalable, feeder-free NK manufacturing process from UDCs

Next, we established a highly efficient, scalable, and feeder-free cell differentiation process for the manufacturing of UDC-NK cells for clinical applications. The overall process consisted of three sequential steps: (1) a 14-day differentiation of CD34^+^ hematopoietic progenitor cells (HPCs) from iPSCs, (2) a 21-day UDC-NK cell differentiation phase, and (3) a 21-day NK activation and expansion phase consisting of three consecutive cycles of 3-day activation followed by 4-day expansion (**Figure 2A**). This 56-day process enabled the generation of sufficient numbers of UDC-NK cells for potential clinical use. In the first step, UDC-iPSCs were seeded into AggreWell™ plates to promote embryoid body (EB) formation and cultured in the presence of a cocktail of cytokines and small molecules for 14 days to induce hematopoietic differentiation toward CD34^+^ HPCs. After HPC differentiation, CD34^+^CD43^+^ cells emerged, followed by the appearance of CD34^+^CD45^+^ cells, consistent with progression toward definitive hematopoiesis (**Supplementary Figure 1A**). In the second step, CD34^+^ HPCs were transferred to culture vessels pre-coated with RetroNectin™ and Delta-like ligand-4 (DLL4) and cultured in the presence of cytokine cocktails and the small molecule UM171. DLL4-mediated Notch signaling has been reported to promote NK lineage commitment (35), while UM171 enhances HPC self-renewal and expansion, thereby improving NK cell yield (30, 31, 36). After 3 weeks of differentiation, the majority of cells (typically 70-90%) exhibited a CD45^+^CD56^+^ NK cell phenotype. In addition, consistent expression of activating receptors, including NKp30 and DNAM-1, was observed (**Supplementary Figure 1B**). In the third step, CD56^+^ UDC-NK cells were harvested and transferred to culture vessels coated with anti-NKp30 and anti-DNAM-1 antibodies and cultured in medium containing IL-15, IL-18, and IL-21 for 3 days to induce activation. Activated UDC-NK cells were subsequently transferred to spinner flasks and cultured in the presence of IL-15 and IL-18, but in the absence of IL-21, for further expansion for 4 days. These activation and expansion cycles were repeated three times to promote NK cell maturation and maximize cell yield. UDC-NK cells consistently expanded by approximately 10-fold during each 7-day activation/expansion cycle, and three consecutive cycles resulted in an overall expansion of >1,000-fold within 21 days (**Figure 2B**). Following three rounds of activation and expansion, the cells exhibited a uniform population with >99% CD45^+^CD3^-^CD56^+^ NK cell phenotype (**Figure 2C**). After cell harvest, UDC-NK cells were cryopreserved for further characterization *in vitro* and *in vivo*. These results demonstrate the establishment of a robust, scalable manufacturing platform for generating large numbers of UDC-NK cells.

**Figure 2:**
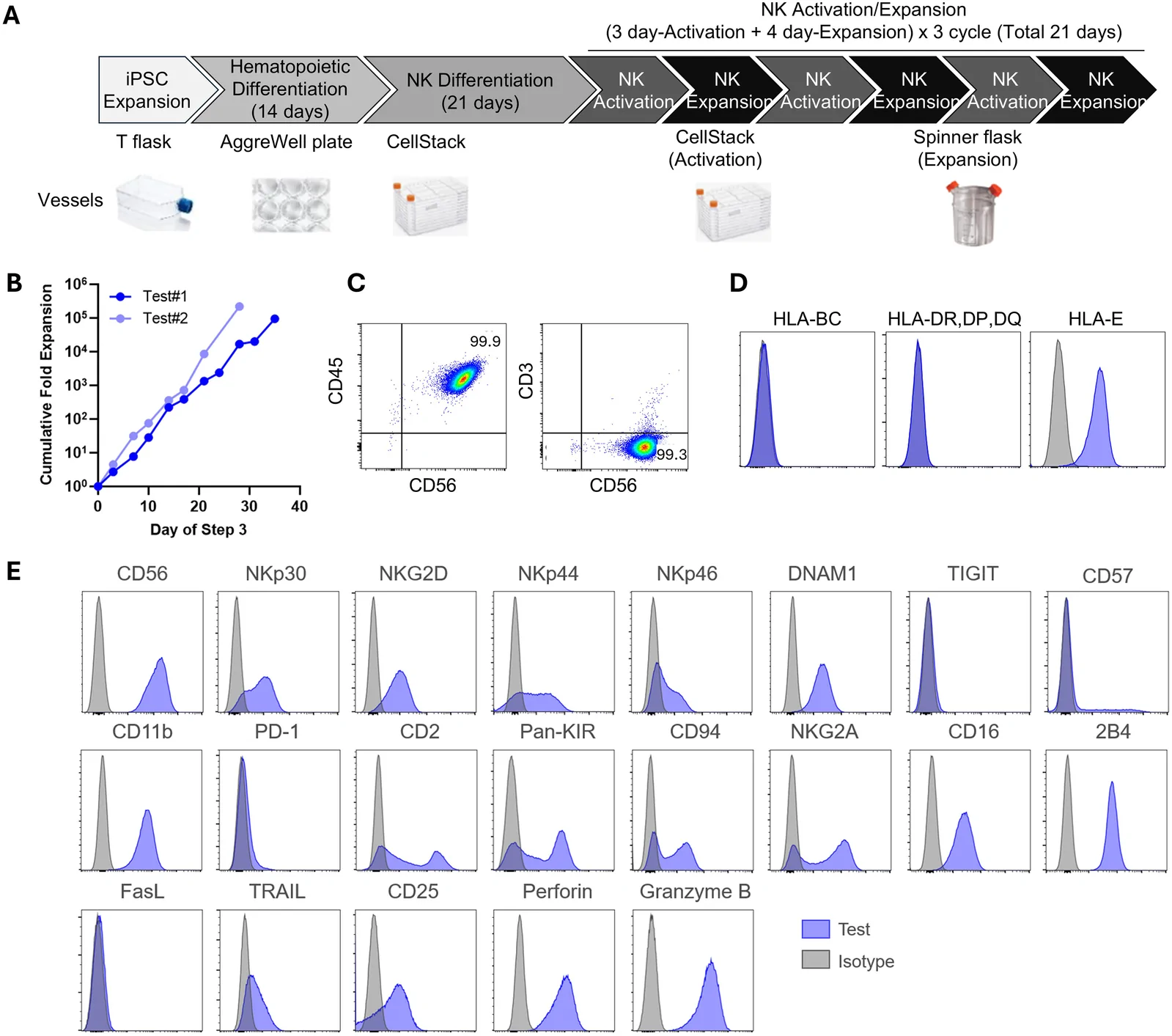
Highly efficient NK cell manufacturing platform from UDC-iPSCs. **(A)** Overview of the UDC-NK cell manufacturing platform, which is composed of 3 steps: (1) 14-day differentiation from iPSCs to Hematopoietic progenitor cells (HPCs), (2) 21-day differentiation from HPCs to immature NK cells, and (3) 21-day serial activation and expansion of immature NK cells to manufacture a large number of highly functional NK cells. **(B)** Continuous expansion of UDC-NK cells by repeating the 7-day NK activation and expansion process. Cumulative fold expansions from two independent manufacturing runs are shown. **(C)** Purity of UDC-NK cells at harvest on day 14 + 21 + 21 by flow cytometry. **(D)** HLA phenotype of UDC-NK cells (blue) compared to isotype controls (gray) by flow cytometry. **(E)** Surface marker phenotype of UDC-NK cells (blue) compared to isotype controls (grey). Cryopreserved UDC-NK cells were thawed, immediately stained, and analyzed by flow cytometry. See **Supplementary Table S1** for antibody details.

We further characterized the phenotype of UDC-NK cells by assessing the expression of key NK cell receptors and cytotoxicity-associated molecules, as well as HLA phenotype by flow cytometry. First, UDC-NK cells retained the engineered genotype introduced at the iPSC stage, as evidenced by sustained expression of HLA-E and the absence of HLA class I and class II molecules (**Figure 2D**). The cells expressed natural cytotoxicity receptors, including NKp30, NKp44, and NKp46, as well as activating receptors such as DNAM-1, NKG2D, CD2, and 2B4. In addition, expression of killer cell immunoglobulin-like receptors (KIRs) was observed, indicating that the UDC-NK cells were matured. Notably, the cells exhibited high levels of cytotoxic effector molecules, including TRAIL, perforin, and granzyme B, consistent with a cytotoxic NK cell phenotype. In contrast, minimal expression of inhibitory receptors such as PD-1 and TIGIT was observed, suggesting a low level of cellular exhaustion (**Figure 2E**). Collectively, these phenotypic data indicate that the established feeder-free manufacturing process enables the robust generation of UDC-NK cells with a characteristic activated immune phenotype.

### 3.3 Comprehensive functional characterization of cryopreserved UDC-NK cells

NK cells possess an intrinsic ability to distinguish tumor cells from healthy cells through a balance of activating and inhibitory receptors, a mechanism known as natural cytotoxicity (8). We first evaluated the natural cytotoxic activity of UDC-NK cells *in vitro*. UDC-NK cells were thawed, washed, and immediately co-cultured with K562 cells, which are highly susceptible to NK cell–mediated lysis and are widely used as target cells, to assess natural cytotoxicity (37). UDC-NK cells exhibited potent cytotoxicity against K562 cells *in vitro* (**Figure 3A**). In addition to natural cytotoxicity, UDC-NK cells were engineered to express high and uniform levels of FCGR3A and were therefore expected to exhibit antibody-dependent cellular cytotoxicity (ADCC) (38). To evaluate ADCC, UDC-NK cells were co-cultured with CD20^+^ B-cell lymphoma Ramos cells in the presence of Rituximab, a therapeutic monoclonal antibody targeting CD20. UDC-NK cells demonstrated strong cytotoxicity against Ramos cells, which was further enhanced in the presence of Rituximab. (**Figure 3B**). Proinflammatory cytokine secretion, including tumor necrosis factor-α (TNF-α) and interferon-γ (IFN-γ), is a key functional property of NK cells that contributes to stimulating both innate and adaptive immune responses. To assess cytokine production, UDC-NK cells were stimulated with K562 cells or CD20^+^ Raji B-cell lymphoma cells in the presence or absence of Rituximab for 24 hours. UDC-NK cells produced minimal amounts of TNF-α and IFN-γ in the absence of stimulation, whereas cytokine secretion was significantly increased upon stimulation with K562 cells or Rituximab-coated Raji cells (**Figures 3C, D**). Serial cytotoxicity is an important characteristic of *in vivo* antitumor efficacy. To assess this property, we established a co-culture system of UDC-NK cells and CD20^+^ Ramos cells in the presence of Rituximab. Notably, UDC-NK cells exhibited persistent serial cytotoxicity against Ramos cells even in the absence of Rituximab and demonstrated more robust serial ADCC activity upon Rituximab treatment during repeated tumor cell challenge (**Figure 3E**). We also assessed the ADCC activity of UDC-NK cells against a panel of CD20^+^ non-Hodgkin B-cell lymphoma cell lines. All tested cell lines uniformly expressed CD20 on their surface with variable density (**Supplementary Figure 2**). Consistent with previous observations, UDC-NK cells exhibited significant cytotoxicity across a broad range of B-cell lymphoma cell lines, indicating robust and reproducible antitumor activity. Importantly, this cytotoxicity was further enhanced in the presence of Rituximab, supporting the ability of UDC-NK cells to mediate ADCC (**Supplementary Figure 3**).

**Figure 3:**
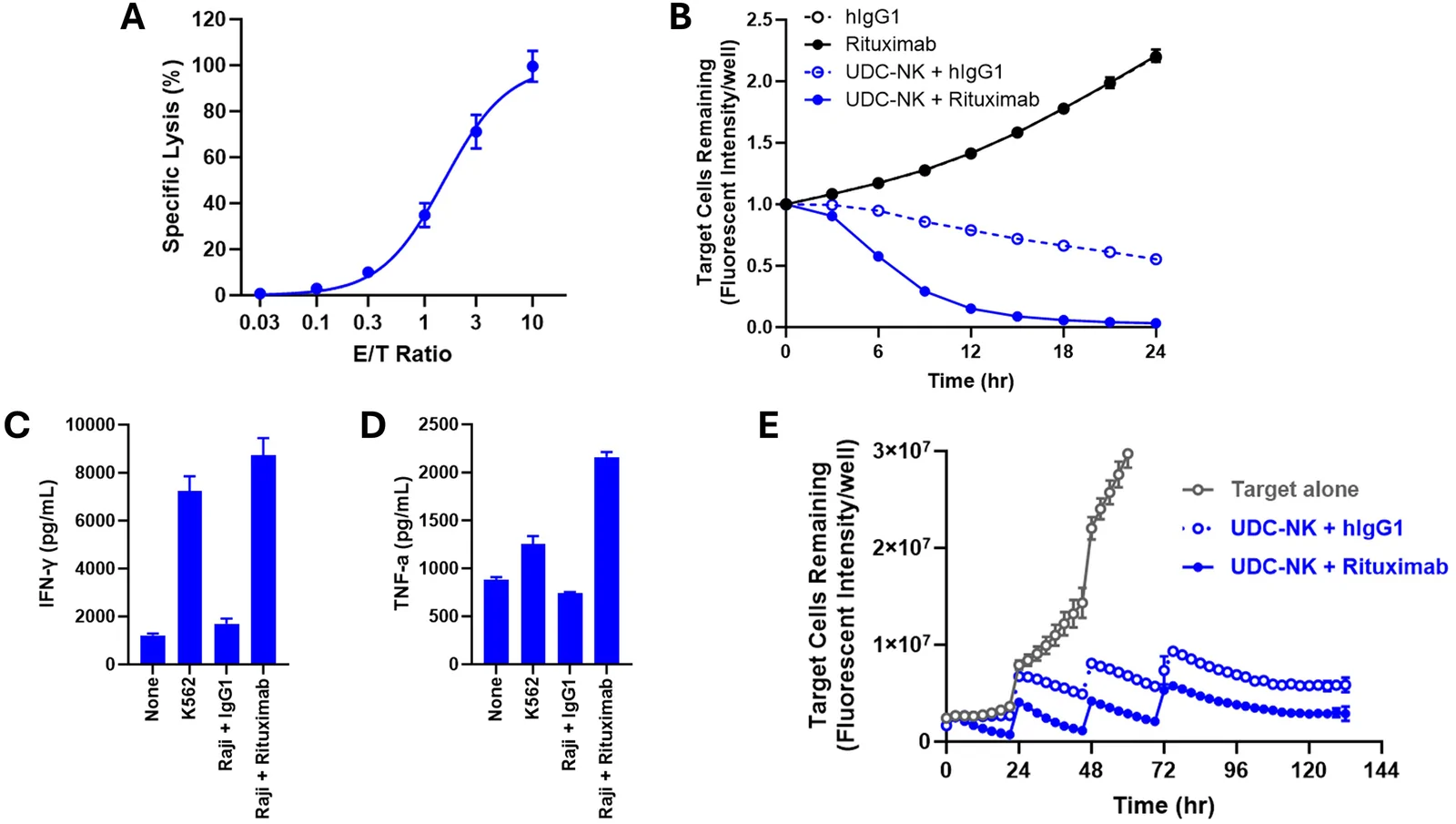
Function of UDC-NK cells *in vitro*. **(A)** 4-hour cytotoxicity of UDC-NK cells against K562 cells. The cytotoxicity was assessed by measuring HiBiT luciferase released from lysed K562 cells. Data are presented as mean ± SD. **(B)** 24-hour ADCC assay with IncuCyte live cell imaging system. UDC-NK cells and NucLight Red–expressing CD20^+^ Ramos cells were co-cultured at an effector-to-target (E:T) ratio of 10:1 in the presence (blue) or absence (dashed blue) of 1 µg/mL Rituximab. The remaining target cells were quantified based on fluorescence intensity. Data are shown as mean ± SD from three independent experiments. Because the Rituximab-only and hIgG1-only groups showed highly similar tumor growth profiles, the hIgG1-only curve may not be fully distinguishable in the graph. **(C, D)** IFN-γ and TNF-α secretion by UDC-NK cells. UDC-NK cells were cocultured with K562 or Raji cells at a 1:1 E:T ratio in the presence or absence of 1 µg/mL Rituximab. After 24 hours of stimulation, culture supernatants were collected for cytokine analysis. Data are shown as mean ± SD from three independent experiments. **(E)** Serial ADCC against CD20^+^ Ramos cells with IncuCyte live cell imaging system. UDC-NK cells and NucLight Red-expressing CD20^+^ Ramos cells were co-cultured at a 10:1 E:T ratio in the presence (blue) or absence (dashed blue) of 1 μg/mL Rituximab. On days 1, 2, and 3 after co-culture initiation, fresh Ramos cells were added together with medium containing IL-15 and Rituximab (blue) or an isotype control (dashed blue) to assess the serial cytotoxic capacity of UDC-NK cells. Data are shown as mean ± SD from three independent experiments.

Collectively, these findings demonstrate that cryopreserved UDC-NK cells exhibit robust natural cytotoxicity, potent and sustained ADCC activity, and inducible proinflammatory cytokine secretion, supporting their potential as an off-the-shelf cell therapy platform for CD20^+^ B-cell malignancies.

### 3.4 Phenotypic and functional change of UDC-NK cells during serial activation/expansion

Next, to assess the potential of our unique NK cell activation and expansion process, which combines anti-NKp30 and anti-DNAM-1 antibodies with a cytokine cocktail, UDC-NK cells were subjected to up to seven consecutive activation/expansion cycles over 49 days, followed by phenotypic and functional characterization. Surprisingly, UDC-NK cells continued expansion during seven rounds of serial expansion, resulting in approximately 10^9^-fold expansion (**Supplementary Figure 4**). Flow cytometric analysis revealed increased expression of DNAM-1, NKG2D, and CD25, whereas TIGIT expression was not detectable throughout the expansion period. Notably, CD2 expression was detected in a minor population after the first round of expansion; however, the frequency of CD2^+^ UDC-NK cells increased over time (**Supplementary Figure 5**). We next assessed the cytotoxicity of UDC-NK cells against K562 cells and found that their activity was maintained over the expansion period (**Supplementary Figure 6A**). Interestingly, serially expanded UDC-NK cells showed a trend toward increased IFN-γ production upon stimulation, while no clear change was observed in TNF-α production (**Supplementary Figure 6B, C**). These results indicate that UDC-NK cells retain their functional competence even after prolonged serial expansion.

### 3.5 Immune evasion of UDC-NK cells from allogeneic T cells and NK cells

UDC-NK cells were engineered by knocking out the *B2M* and *RFXANK* genes to eliminate surface expression of HLA class I and class II molecules, thereby preventing allogeneic T cell–mediated recognition. In addition, HLA-E was knocked in to enable UDC-NK cells to evade allogeneic NK cell–mediated cytotoxicity¹^8^. To evaluate the immune evasion of UDC-NK cells, we assessed their susceptibility to allogeneic NK cell–mediated cytotoxicity. Notably, UDC-NK cells exhibited complete resistance to NK cell–mediated lysis, whereas B2M-knockout cells lacking HLA-E expression remained highly susceptible (**Figure 4A**). Importantly, UDC-NK cells did not induce proliferation of either CD4^+^ or CD8^+^ T cells, indicating immune evasion capacity of UDC-NK cells, whereas NK cells differentiated from WT iPSCs stimulated robust proliferation of allogeneic T cells (**Figure 4B**). Collectively, these results demonstrate that UDC-NK cells effectively evade both allogeneic T cell– and NK cell–mediated immune responses *in vitro*, supporting their potential to improve persistence and enhance antitumor efficacy *in vivo*.

**Figure 4:**
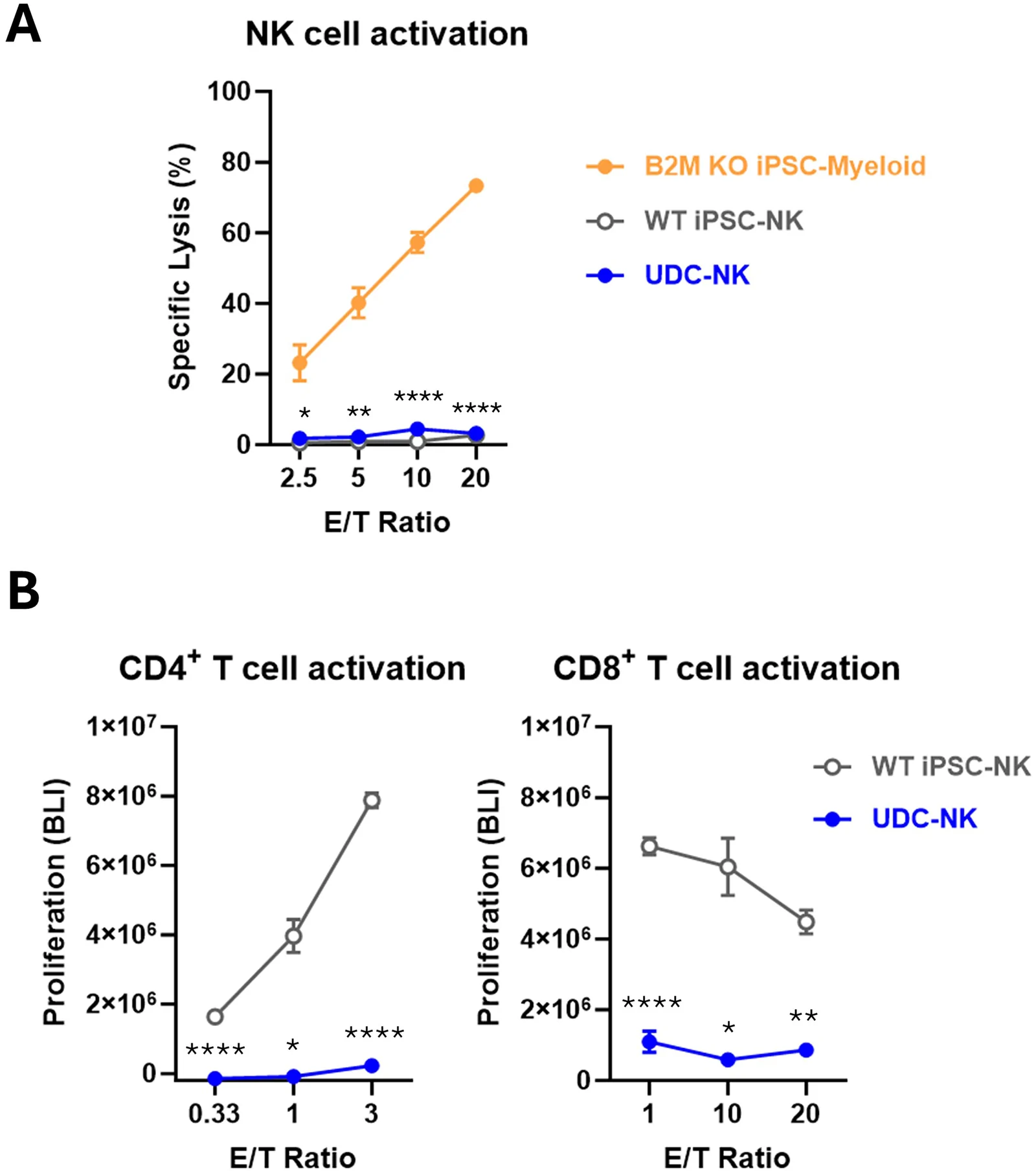
Immune evasion of UDC-NK cells from allogeneic T cells and NK cells. **(A)** UDC-NK cells (blue), WT iPSC-NK cells (gray), and B2M knockout iPSC-derived myeloid cells (orange) were labeled with bis(acetoxymethyl) 2,2′:6′,2″-terpyridine-6,6″-dicarboxylate (BATDA) and used as targets. BATDA-labeled target cells were co-cultured with peripheral blood–derived NK cells for 2 hours. Target cell lysis was quantified by measuring 2,2′:6′,2″-terpyridine-6,6″-dicarboxylic acid (TDA), a fluorescent ligand released upon lysis of targets, using a DELFIA cytotoxicity assay. Data are presented as mean ± SD from three independent experiments. **(B)** UDC-NK cells (blue) and WT iPSC-NK cells (gray) were co-cultured with CD4^+^ or CD8^+^ T cells isolated from the peripheral blood of healthy donors for 3 days. T cell proliferation was assessed using a CellTiter-Glo cell viability assay. Data are presented as mean ± SD from three independent experiments. *P < 0.05, **P < 0.01, and ****P < 0.0001.

### 3.6 Introduction of a Herpes Simplex Virus Thymidine Kinase (HSV-TK) safety switch

Adoptive cell therapies, such as autologous CAR-T cells, have demonstrated remarkable efficacy in hematological malignancies and are now widely used in clinical practice. However, the management of severe adverse events, including cytokine release syndrome and immune effector cell–associated neurotoxicity syndrome, remains a major clinical challenge (39). To mitigate potential safety risks associated with excessive immune activation, UDC-NK cells were engineered to express two copies of the herpes simplex virus-derived thymidine kinase (HSV-TK) genes as a safety switch. This system can be pharmacologically activated by ganciclovir, a small-molecule antiviral agent commonly used to treat cytomegalovirus infections. Upon ganciclovir administration, HSV-TK-expressing cells are selectively eliminated, enabling controlled removal of UDC-NK cells in the event of adverse reactions (37, 38, 40, 41). To evaluate the functionality of this safety switch, UDC-NK cells and control iPSC-derived NK cells differentiated from a wild-type iPSC clone were cultured in the presence of increasing concentrations of ganciclovir, and cell viability was assessed after 4 days of treatment. No significant cytotoxicity was observed in the absence of ganciclovir. UDC-NK cells expressing HSV-TK were efficiently eliminated in a dose-dependent manner upon ganciclovir treatment, whereas wild-type iPSC-NK cells showed no significant reduction in viability (**Figure 5**). Notably, UDC-NK cells were completely eliminated at clinically relevant ganciclovir concentrations (1-100 µM) (39). These results demonstrate that the HSV-TK safety switch is functional in UDC-NK cells and provides a mechanism for selective cell ablation, thereby supporting the safety of the UDC-NK cell platform.

**Figure 5:**
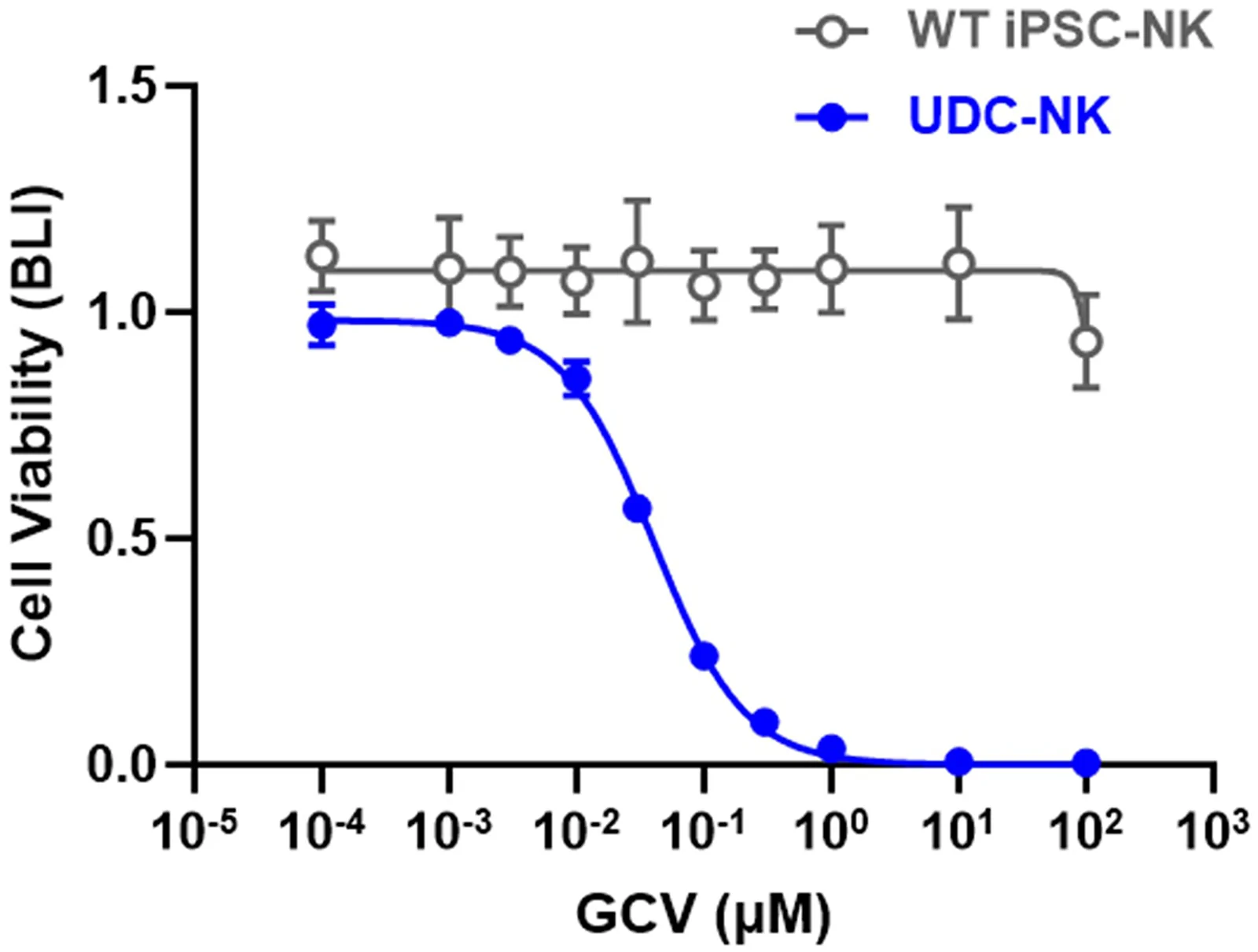
Herpes simplex virus thymidine kinase (HSV-TK)-driven safety switch. UDC-NK cells with the HSV-TK gene (blue) and iPSC-NK cells differentiated from WT iPSC (grey) were cultured in the presence of a titrated concentration of ganciclovir for 4 days. After culture, cell viability was measured by the CellTiter-Glo cell viability assay. Data are presented as mean ± SD from three independent experiments.

### 3.7 Efficacy of UDC-NK cells in combination with rituximab in B-cell lymphoma xenograft models

Following our *in vitro* characterization, we next investigated the *in vivo* persistence and safety profile of UDC-NK cells using NOG-IL-15 mice, an immunodeficient mouse strain transgenic for human IL-15 to support human NK cell persistence. We thawed cryopreserved UDC-NK cells and immediately administered 1.5x10^7^ cells per mouse. In the flow cytometric analysis, UDC-NK cells were detected most frequently on day 3, and their frequency decreased by day 7 after administration (**Supplementary Figure 7A**). The mice exhibited transient body weight loss but fully recovered by day 7, with no obvious clinical signs of deterioration (**Supplementary Figure 7B**).

Finally, to evaluate the antitumor activity of cryopreserved UDC-NK cells *in vivo*, we established disseminated lymphoma xenograft models in NOGF-IL-15 mice, an immunodeficient strain lacking surface Fc receptor expression and constitutively expressing human IL-15. The absence of murine Fc receptors eliminates ADCC and antibody-dependent cellular phagocytosis (ADCP) mediated by mouse effector cells, thereby enabling selective evaluation of ADCC mediated by UDC-NK cells *in vivo*. In addition, systemic expression of human IL-15 supports NK cell persistence (32, 42). CD20^+^ B lymphoma Raji cells were intravenously injected into NOGF-IL-15 mice to establish disseminated lymphoma. One day after tumor inoculation, mice were treated with Rituximab alone or in combination with cryopreserved UDC-NK cells administered twice weekly for three weeks (**Figure 6A**). Rituximab monotherapy resulted in moderate inhibition of tumor progression compared with untreated controls. In contrast, the combination of UDC-NK cells and Rituximab showed approximately 99% suppression of tumor progression (**Figure 6B**). Consistent with this marked antitumor activity, mice treated with the combination of UDC-NK cells and Rituximab exhibited a significant survival advantage compared with all other treatment groups (**Figure 6C**). Representative IVIS images demonstrated effective suppression of tumor growth in the UDC-NK plus Rituximab group, consistent with the reduced tumor burden and improved survival observed in treated mice. (**Figure 6D**).

**Figure 6:**
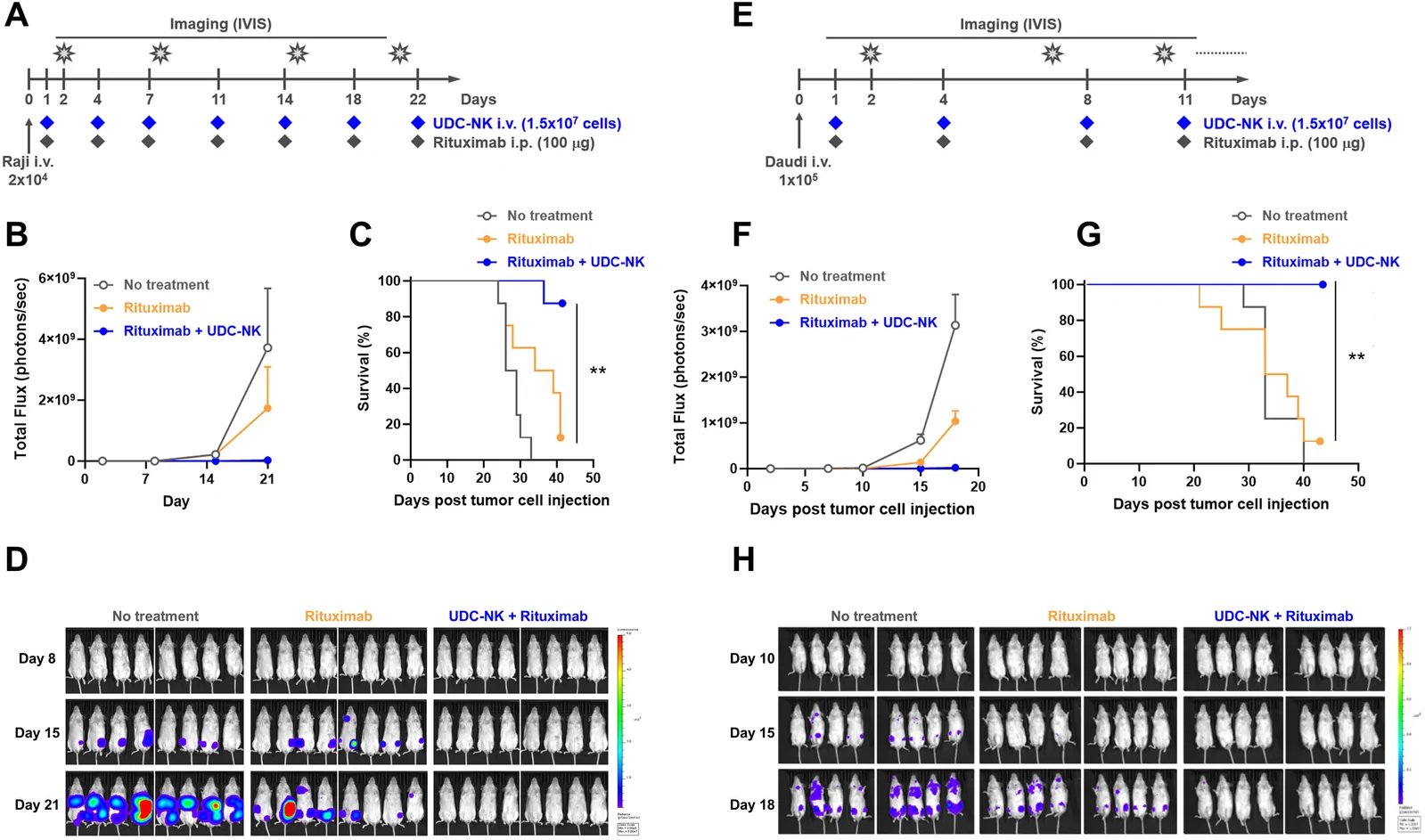
Efficacy of cryopreserved UDC-NK cells in combination with Rituximab in disseminated B-cell lymphoma mouse models. **(A)** Schematic of a disseminated Raji B lymphoma mouse model. NOGF-IL-15 mice were intravenously inoculated with 2 x 104 Luciferase-expressing Raji cells. On day 1, mice were left untreated or treated with Rituximab (100 μg) alone or in combination with cryopreserved UDC-NK cells (1.5x107), administered twice weekly for 3 weeks. Tumor progression was monitored with IVIS imaging. **(B)** Time course of Raji tumor burden quantified by IVIS imaging. Data are presented as mean ± SEM, (n=8). Statistical analysis was performed using two-way repeated measures ANOVA followed by multiple comparisons test. **(C)** Kaplan-Meier survival curves of the experimental groups. Statistical significance was determined using the log-rank (Mantel–Cox) test. **(D)** Representative IVIS bioluminescence images of Raji tumor-bearing mice from the indicated treatment groups at the indicated time points. **(E)** Schematic of a disseminated Daudi B lymphoma mouse model. NOGF-IL-15 mice were intravenously inoculated with 105 Luciferase-expressing Daudi cells. On day 1, mice were left untreated or treated with Rituximab (100 μg) alone or in combination with cryopreserved UDC-NK cells (1.5x107), administered twice weekly for 2 weeks. Tumor progression was monitored with IVIS imaging. **(F)** Time course of Daudi tumor burden quantified by IVIS imaging. Data are presented as mean ± SEM, (n=8). Statistical analysis was performed using two-way repeated measures ANOVA followed by multiple comparisons test. **(G)** Kaplan-Meier survival curves of the experimental groups. Statistical significance was determined using the log-rank (Mantel–Cox) test. **(H)** Representative IVIS bioluminescence images of Daudi tumor-bearing mice from the indicated treatment groups at the indicated time points. **P < 0.01.

We also employed a disseminated B-cell lymphoma model using CD20^+^ Daudi cells to evaluate the *in vivo* efficacy of cryopreserved UDC-NK cells (**Figure 6E**). In this context, repeated administration of UDC-NK cells in combination with Rituximab resulted in robust antitumor activity, achieving 99% tumor growth inhibition (**Figure 6F**). Furthermore, the combination significantly improved overall survival in treated mice (**Figure 6G**). Representative IVIS imaging further confirmed effective suppression of tumor progression in the UDC-NK plus rituximab group, consistent with the tumor burden and survival data (**Figure 6H**). Collectively, these results demonstrate that UDC-NK cells exert potent antitumor activity in combination with Rituximab through the ADCC mechanism *in vivo*. These findings support the potential of UDC-NK cells as an effective off-the-shelf cellular immunotherapy in combination with therapeutic antibodies for the treatment of hematological malignancies (43).

## 4 Discussion

Allogeneic NK cell therapies are emerging as a promising alternative to T cell-based immunotherapies due to their intrinsic ability to recognize and eliminate malignant cells without prior antigen sensitization, as well as their favorable safety profile (8, 44, 45). In this study, we developed a universal donor iPSC-derived NK cell (UDC-NK) cell platform that integrates multiple genetic modifications to enhance both therapeutic efficacy and safety, establishing a novel approach to generating immune-evasive NK cells with a favorable safety profile. Using a novel feeder-free and scalable manufacturing process, we generated highly functional NK cells exhibiting canonical NK cell phenotypes, potent natural cytotoxicity, and robust antibody-dependent cellular cytotoxicity (ADCC). Notably, UDC-NK cells demonstrated strong antitumor activity in xenograft lymphoma models, particularly in combination with Rituximab, leading to near-complete suppression of tumor progression and significantly prolonged survival.

A notable aspect of this study is the development of a feeder-free manufacturing process that enables robust expansion of genetically engineered iPSC-derived NK cells while maintaining NK-cell purity and functionality. Many previously reported iPSC-NK manufacturing approaches have relied on feeder-cell-based expansion systems, which may add complexity to large-scale clinical manufacturing. In contrast, our novel feeder-free activation and expansion process utilizing agonists of NK-cell activating receptors together with a cytokine cocktail supported extensive expansion and generated large numbers of UDC-NK cells while preserving NK-cell phenotype and cytotoxic function. These findings suggest that this platform may facilitate scalable and standardized manufacturing of off-the-shelf NK-cell products. Furthermore, the sequential emergence of CD34^+^CD43^+^/CD34^+^CD45^+^ hematopoietic progenitors and CD45^+^CD56^+^ NK cells may provide useful in-process checkpoints for monitoring differentiation progression and manufacturing consistency. Future studies should evaluate whether these markers can serve as predictive quality attributes for clinical-scale production.

A central component of this platform is the use of a multi-gene-edited universal donor iPSC line engineered to evade host immune rejection (19, 24, 44). Disruption of *B2M* and *RFXANK* eliminates surface expression of HLA class I and II molecules, thereby preventing recognition by allogeneic T cells, while the introduction of a single-chain HLA-E construct provides protection against NK cell–mediated cytotoxicity (19). Consistent with this design, UDC-NK cells did not activate allogeneic CD4^+^ or CD8^+^ T cells and were resistant to cytotoxicity mediated by allogeneic PBMC-derived NK cells *in vitro*. Consequently, UDC-NK cells represent a novel class of engineered cellular therapeutics that integrate immune evasion strategies with a built-in safety switch system. Recently, novel UDC technologies utilizing CD47 overexpression and Autoimmune Defense Receptors (ADRs) targeting 4-1BB have been reported to further enhance immune evasion (24, 25). Accordingly, the UDC platform described here could be further enhanced by combining HLA editing with these additional gene-engineering strategies. Although the immune-evasive properties of UDC-NK cells have so far been evaluated only *in vitro*, further validation in robust *in vivo* models will be necessary to confirm their translational potential. UDC-NK cells were engineered to express herpes simplex virus thymidine kinase (HSV-TK) safety switch (39). The safety switch is a well-characterized and clinically validated system that enables the selective pharmacological elimination of engineered cells upon ganciclovir administration (37, 43). Activation of this safety switch by ganciclovir resulted in efficient elimination of UDC-NK cells *in vitro*, providing an additional layer of control and may help manage potential adverse events associated with cell therapy. Importantly, the functionality of this safety switch has only been demonstrated *in vitro*. Future studies will be required to assess the kinetics, completeness, and durability of UDC-NK cell elimination following ganciclovir administration under clinically relevant *in vivo* conditions. Such studies will be important to further establish the translational utility of the HSV-TK safety switch for the management of potential cell therapy-related toxicities.

Another important feature of UDC-NK cells is the uniform expression of the high-affinity *FCGR3A* (F158V) variant, which enhances ADCC (21). NK cell-mediated ADCC plays a critical role in the therapeutic activity of many clinically approved monoclonal antibodies, including Rituximab (43). Consistent with this mechanism, UDC-NK cells demonstrated robust ADCC against multiple CD20-positive lymphoma cell lines in the presence of Rituximab, while retaining natural cytotoxic activity in the absence of antibody stimulation. The ability to mediate both natural cytotoxicity and antibody-dependent killing may provide complementary mechanisms of tumor control and enhance the therapeutic potential of this engineered NK cell platform. Importantly, the enhanced cytotoxic activity observed *in vitro* translated into substantial therapeutic benefits *in vivo*. In Fc receptor-deficient NOGF-IL-15 mice, which enable selective evaluation of ADCC mediated by administered NK cells, the combination of UDC-NK cells and Rituximab resulted in near-complete suppression of tumor progression and significantly improved survival compared with Rituximab monotherapy in Raji and Daudi B cell lymphoma disseminated mouse models. These findings support the concept that engineering NK cells to express high-affinity Fc receptors can enhance the efficacy of antibody-based cancer therapies (43). Such combination approaches may represent a promising strategy to improve the clinical activity of monoclonal antibodies in hematological malignancies. However, a limitation of the present study is that ADCC activity was not directly compared with that of CD16-unmodified iPSC-derived NK cells. Therefore, although robust ADCC activity was demonstrated, the extent to which FCGR3A (F158V) engineering contributed to the enhanced ADCC phenotype could not be precisely determined. Furthermore, the relative contributions of natural cytotoxicity and ADCC to the observed antitumor efficacy remain unclear because UDC-NK monotherapy was not systematically evaluated *in vivo*. Future studies incorporating appropriate comparator NK-cell products and UDC-NK monotherapy groups will be necessary to address these questions.

More broadly, the present study did not include direct side-by-side comparisons between UDC-NK cells and primary NK cells or unedited iPSC-derived NK cells under identical manufacturing and assay conditions. Future comparative studies performed under standardized manufacturing and assay conditions will be important to further define the relative advantages of the UDC-NK platform.

In addition, further optimization of this platform may enhance its therapeutic potential. For example, combining UDC-NK cells with additional antibody-based therapeutics, such as PD-1 immune checkpoint inhibitors, may further improve antitumor responses. Moreover, the gene-editing strategy employed in this study could be extended to incorporate additional modifications to enhance NK cell cytotoxicity, persistence, trafficking, and resistance to immunosuppressive signals within the tumor microenvironment (12, 22, 23, 42, 46). Such next-generation engineering approaches may include the introduction of chimeric antigen receptors (CARs), expression of membrane-bound cytokines such as IL-15 to promote persistence (17, 22, 23), or disruption of inhibitory pathways such as CISH to augment NK cell activation (47).

In conclusion, we developed a universal donor iPSC-derived NK cell platform that integrates immune evasion, enhanced ADCC capability, and an inducible safety switch within a feeder-free, scalable manufacturing framework. UDC-NK cells manufactured from multiple gene-edited UDC-iPSCs exhibit potent antitumor activity both *in vitro* and *in vivo*, particularly in combination with antibody-based therapies such as Rituximab. These findings support the potential of UDC-NK cells as a next-generation, off-the-shelf NK cell therapy for hematologic malignancies and potentially solid tumors.

## Data Availability

The original contributions presented in the study are included in the article/**Supplementary Material**. Further inquiries can be directed to the corresponding author.

## References

[1] June CH O’Connor RS Kawalekar OU Ghassemi S Milone MC . CAR T cell immunotherapy for human cancer. Science. (2018) 359:1361–5. doi: 10.1126/science.aar6711 29567707

[2] Maude SL Laetsch TW Buechner J Rives S Boyer M Bittencourt H . Tisagenlecleucel in children and young adults with B-cell lymphoblastic leukemia. N Engl J Med. (2018) 378:439–48. doi: 10.1056/NEJMoa1709866 29385370 PMC5996391

[3] Newick K O’Brien S Moon E Albelda SM . CAR T cell therapy for solid tumors. Annu Rev Med. (2017) 68:139–52. doi: 10.1146/annurev-med-062315-120245 27860544

[4] Zhang Y Qin D Shou AC Liu Y Wang Y Zhou L . Exploring CAR-T cell therapy side effects: mechanisms and management strategies. J Clin Med. (2023) 12:6124. doi: 10.3390/jcm12196124 37834768 PMC10573998

[5] Long EO Kim HS Liu D Peterson ME Rajagopalan S . Controlling natural killer cell responses: integration of signals for activation and inhibition. Annu Rev Immunol. (2013) 31:227–58. doi: 10.1146/annurev-immunol-020711-075005 23516982 PMC3868343

[6] Lanier LL . NK cell recognition. Annu Rev Immunol. (2005) 23:225–74. doi: 10.1146/annurev.immunol.23.021704.115526 15771571

[7] Karlhofer FM . MHC class I alloantigen specificity of Ly-49^+^ IL-2-activated natural killer cells. Nature. (1992) 358:66–70. doi: 10.1038/358066a0 1614533

[8] Vivier E Raulet DH Moretta A Caligiuri MA Zitvogel L Lanier LL . Innate or adaptive immunity? The example of natural killer cells. Science. (2011) 331:44–9. doi: 10.1126/science.1198687 21212348 PMC3089969

[9] Ravetch JV Bolland S . IgG fc receptors. Annu Rev Immunol. (2001) 19:275–90. doi: 10.1146/annurev.immunol.19.1.275 11244038

[10] Nimmerjahn F Ravetch JV . Fcγ receptors as regulators of immune responses. Nat Rev Immunol. (2008) 8:34–47. doi: 10.1038/nri2206 18064051

[11] Rezvani K Rouce R . The application of natural killer cell immunotherapy for the treatment of cancer. Nat Rev Immunol. (2015) 15:341–54. doi: 10.1038/nri3803 26635792 PMC4648067

[12] Liu E Marin D Banerjee P Macapinlac HA Thompson P Basar R . Use of CAR-transduced natural killer cells in CD19-positive lymphoid tumors. N Engl J Med. (2020) 382:545–53. doi: 10.1056/NEJMoa1910607 32023374 PMC7101242

[13] Miller JS Soignier Y Panoskaltsis-Mortari A McNearney SA Yun GH Fautsch SK . Successful adoptive transfer and *in vivo* expansion of human haploidentical NK cells in patients with cancer. Blood. (2005) 105:3051–7. doi: 10.1182/blood-2004-07-2974 15632206

[14] Takahashi K Tanabe K Ohnuki M Narita M Ichisaka T Tomoda K . Induction of pluripotent stem cells from adult human fibroblasts by defined factors. Cell. (2007) 131:861–72. doi: 10.1016/j.cell.2007.11.019 18035408

[15] Knorr DA Ni Z Hermanson D Hexum MK Bendzick L Cooper LJ . Clinical-scale derivation of natural killer cells from human pluripotent stem cells for cancer therapy. Stem Cells Transl Med. (2013) 2:274–83. doi: 10.5966/sctm.2012-0084 23515118 PMC3659832

[16] Li Y Hermanson DL Moriarity BS Kaufman DS . Human iPSC-derived natural killer cells engineered with chimeric antigen receptors enhance anti-tumor activity. Cell Stem Cell. (2018) 23:181–92. doi: 10.1016/j.stem.2018.06.002 30082067 PMC6084450

[17] Cichocki F Goodridge JP Bjordahl R Mahmood S Davis ZB Gaidarova S . Dual antigen-targeted off-the-shelf NK cells show durable response and prevent antigen escape in lymphoma and leukemia. Blood. (2022) 140:2451–62. doi: 10.1182/blood.2021015184 35917442 PMC9918847

[18] Riolobos L Hirata RK Turtle CJ Wang PR Gornalusse GG Zavajlevski M . HLA engineering of human pluripotent stem cells. Mol Ther. (2013) 21:1232–41. doi: 10.1038/mt.2013.59 23629003 PMC3677304

[19] Gornalusse GG Hirata RK Funk SE Riolobos L Lopes VS Manske G . HLA-E–expressing pluripotent stem cells escape allogeneic T cell and NK cell responses. Nat Biotechnol. (2017) 35:765–72. doi: 10.1038/nbt.3860 28504668 PMC5548598

[20] Rong Z Wang M Hu Z Stradner M Zhu S Kong H . An effective approach to prevent immune rejection of human ESC-derived allografts. Cell Stem Cell. (2014) 14:121–30. doi: 10.1016/j.stem.2013.11.014 24388175 PMC4023958

[21] Koene HR Kleijer M Algra J Roos D von dem Borne AE de Haas M . FcγRIIIa-158V/F polymorphism influences IgG-mediated NK cell cytotoxicity. Blood. (1997) 90:1109–14. doi: 10.1182/blood.V90.3.1109 9242542

[22] Ghobadi A Bachanova V Patel K Park JH Flinn I Riedell PA . Induced pluripotent stem-cell-derived CD19-directed chimeric antigen receptor natural killer cells in B-cell lymphoma: a phase 1, first-in-human trial. Lancet. (2025) 405:127–36. doi: 10.1016/S0140-6736(24)02462-0 39798981 PMC11827677

[23] Hall MS Manor O Campion L Bullaughey C Morse BA Gurung B . (2024). “ Integrated multi-functional analysis identifies CNTY-101, a clinical-stage iPSC-derived CAR-NK cell therapy incorporating immune-evasion engineering, IL-15 support, and a safety switch”, in: ASGCT Annual Meeting.

[24] Deuse T Hu X Gravina A Wang D Tediashvili G De C . Genome-engineered T cells evade allogeneic rejection in nonhuman primates. Nat Biotechnol. (2021) 39:481–90. doi: 10.1038/s41587-021-00844-537880705

[25] Mamonkin M Mukherjee M Srinivasan M Sharma S Gomes-Silva D Mo F . Engineered off-the-shelf therapeutic T cells resist host immune rejection. Nat Biotechnol. (2021) 39:56–63. doi: 10.1038/s41587-020-0601-5 32661440 PMC7854790

[26] Bonini C Ferrari G Verzeletti S Servida P Zappone E Ruggieri L . HSV-TK gene transfer into donor lymphocytes for control of allogeneic graft-versus-leukemia. Science. (1997) 276:1719–24. doi: 10.1126/science.276.5319.1719 9180086

[27] Bordignon C Bonini C Verzeletti S Nobili N Maggioni D Traversari C . Gene therapy in peripheral blood lymphocytes and bone marrow for control of graft-versus-host disease. Science. (1995) 270:470–5. doi: 10.1126/science.270.5238.470

[28] Di Stasi A Tey SK Dotti G Fujita Y Kennedy-Nasser A Martinez C . Inducible apoptosis as a safety switch for adoptive cell therapy. N Engl J Med. (2011) 365:1673–83. doi: 10.1056/NEJMoa1106152 22047558 PMC3236370

[29] Astellas Pharma Inc . Engineered natural killer cells and related methods. (2024).

[30] Fares I Chagraoui J Gareau Y Gingras S Ruel R Mayotte N . Pyrimidoindole derivatives are agonists of human hematopoietic stem cell self-renewal. Science. (2014) 345:1509–12. doi: 10.1126/science.1256337 25237102 PMC4372335

[31] Cohen S Roy J Lachance S Delisle JS Marinier A Busque L . Umbilical cord blood transplantation using ex vivo expanded stem cells with UM171: a phase I–II clinical trial. Lancet Haematol. (2020) 7:e134–45. doi: 10.1016/S2352-3026(19)30202-9 31704264

[32] Katano I Ito R Kawai K Takahashi T . Improved detection of *in vivo* human NK cell-mediated antibody-dependent cellular cytotoxicity using a novel NOG-fcγR-deficient human IL-15 transgenic mouse. Front Immunol. (2020) 11:532684. doi: 10.3389/fimmu.2020.532684 33117338 PMC7577188

[33] van Wilgenburg B Browne C Vowles J Cowley SA . Efficient, Long term production of monocyte-derived macrophages from human pluripotent stem cells under partly-defined and fully-defined conditions. PloS One. (2013) 8:e71098. doi: 10.1371/journal.pone.0071098 23951090 PMC3741356

[34] Khan IF Hirata RK Russell DW . AAV-mediated gene targeting methods for human cells. Nat Protoc. (2011) 6:482–501. doi: 10.1038/nprot.2011.301 21455185 PMC3739714

[35] Jaleco AC Neves H Hooijberg E Gameiro P Clode N Haury M . Human CD34^+^ cells differentiate into NK cells in the thymus. J Exp Med. (2001) 194:991–1002. doi: 10.1084/jem.194.7.991 11581320 PMC2193482

[36] Orkin SH Zon LI . Hematopoiesis: an evolving paradigm for stem cell biology. Cell. (2008) 132:631–44. doi: 10.1016/j.cell.2008.01.025 18295580 PMC2628169

[37] Kärre K Ljunggren HG Piontek G Kiessling R . Selective rejection of H-2-deficient lymphoma variants suggests alternative immune defence strategy. Nature. (1986) 319:675–8. doi: 10.1038/319675a0 3951539

[38] Weiner GJ . Building better monoclonal antibody-based therapeutics. Nat Rev Cancer. (2015) 15:361–70. doi: 10.1038/nrc3930 25998715 PMC4491443

[39] Traversari C Marktel S Magnani Z Mangia P Russo V Ciceri F . The potential of the HSV-TK suicide gene system for the control of alloreactive T cells. Blood. (2007) 109:4708–17. doi: 10.1182/blood-2006-10-049221 17327417

[40] Liang Q Monetti C Shutova MV Neely EJ Hacibekiroglu S Yang H . Linking a cell-division gene and a suicide gene to define and improve cell therapy safety. Nature. (2018) 563:701–4. doi: 10.1038/s41586-018-0733-7 30429614

[41] Waldmann TA . The biology of interleukin-15: implications for cancer therapy and immunotherapy. Nat Rev Immunol. (2006) 6:595–601. doi: 10.1038/nri1908 16868550

[42] Miller JS Lanier LL . Natural killer cells in cancer immunotherapy. Annu Rev Cancer Biol. (2019) 3:77–103. doi: 10.1146/annurev-cancerbio-030518-05565341139587

[43] Cartron G Dacheux L Salles G Solal-Céligny P Bardos P Colombat P . Therapeutic activity of humanized anti-CD20 monoclonal antibody and polymorphism in IgG Fc receptor FcγRIIIa gene. Blood. (2002) 99:754–8. doi: 10.1182/blood.V99.3.754 11806974

[44] Deuse T Hu X Gravina A Wang D Tediashvili G De C . Hypoimmunogenic pluripotent stem cells induce less immune response and are tolerated in allogeneic recipients. Nat Biotechnol. (2019) 37:252–8. doi: 10.1038/s41587-019-0016-3 30778232 PMC6419516

[45] Ciceri F Bonini C Stanghellini MTL Bondanza A Traversari C Salomoni M . Infusion of suicide gene–modified donor lymphocytes after family haploidentical hematopoietic stem-cell transplantation. Lancet Oncol. (2009) 10:489–500. doi: 10.1016/S1470-2045(09)70074-9 19345145

[46] Daher M Rezvani K . Next generation natural killer cells for cancer immunotherapy: the promise of genetic engineering. Curr Opin Immunol. (2018) 51:146–53. doi: 10.1016/j.coi.2018.03.013 29605760 PMC6140331

[47] Delconte RB Kolesnik TB Dagley LF Rautela J Shi W Putz EM . CIS is a potent checkpoint in NK-cell-mediated tumor immunity. Nat Immunol. (2016) 17:816–24. doi: 10.1038/ni.3470 27213690

