# Supplementary material for: Immune-evading universal donor iPSC-derived natural killer cells enable off-the-shelf cancer immunotherapy
Source: Front Immunol. 2026 Oct 29;17:1938206. doi: 10.3389/fimmu.2026.1938206 (PMC13754875; doi:10.3389/fimmu.2026.1938206)
Supplement: Supplementary file 1 [file SupplementaryFile1.pdf]

SUPPLEMENTARY TABLE 1

Target	Fluorochrome	Clone	Isotype	Company	Catalog Number
OCT3/4	PerCP-Cy5.5	40/Oct-3	Mouse IgG1	BD Biosciences	560794
SOX2	Alexa Fluor 647	245610	Mouse IgG2a	BD Biosciences	560294
SSEA-4	PE	MC-813-71	Mouse IgG3	BioLegend	330405
Isotype Control	PerCP-Cy5.5	MOPC-21	Mouse IgG1	BD Biosciences	552834
Isotype Control	Alexa Fluor 647	MOPC-173	Mouse IgG2a	BD Biosciences	565365
Isotype Control	PE	MG3-35	Mouse IgG3	BioLegend	401319
CD45	FITC	2D1	Mouse IgG1	BioLegend	368508
CD56	PE-Cy7	5.1H11	Mouse IgG1	BioLegend	362510
NKG2D	APC	1D11	Mouse IgG1	BioLegend	320808
DNAM-1	BV421	11A8	Mouse IgG1	BioLegend	338332
NKp46	BV711	9E2	Mouse IgG1	BioLegend	331936
NKp30	PE	P30-15	Mouse IgG1	BioLegend	325208
NKp44	APC-Cy7	P44-8	Mouse IgG1	BioLegend	325214
CD57	APC	QA17A04	Mouse IgG1	BioLegend	393306
CD2	BV421	RPA-2.10	Mouse IgG1	BioLegend	300230
PD-1	BV711	NAT105	Mouse IgG1	BioLegend	367428
TIGIT	PE	TgMab-2	Mouse IgG1	BD Biosciences	568672
CD11b	APC-Cy7	ICRF44	Mouse IgG1	BioLegend	301342
CD94	APC	DX22	Mouse IgG1	BioLegend	305508
NKG2A	BV421	S19004C	Mouse IgG1	BioLegend	375140
CD16	BV711	3G8	Mouse IgG1	BioLegend	302044
CD158a,h	PE	11PB6	Mouse IgG1	Miltenyi Biotec	130-099-209
CD158b	PE	CH-L	Mouse IgG2b	BD Biosciences	561723
CD158d	PE	mAb 33	Mouse IgG1	BioLegend	347006
CD158e1	PE	DX9	Mouse IgG1	BD Biosciences	555967
2B4	APC-Cy7	C1.7	Mouse IgG1	BioLegend,	329518
Fas ligand	APC	NOK-1	Mouse IgG1	BioLegend	306422
Perforin	BV421	B-D48	Mouse IgG1	BioLegend	353307
TRAIL	BV711	RIK-2	Mouse IgG1	BD Biosciences	743722
Granzyme B	PE	GB11	Mouse IgG1	BD Biosciences	561142
CD25	APC-Cy7	M-A251	Mouse IgG1	BioLegend	356122
Isotype control	FITC	MOPC-21	Mouse IgG1	BD Biosciences	556649
Isotype control	APC	MOPC-21	Mouse IgG1	BD Biosciences	550854
Isotype control	BV421	X40	Mouse IgG1	BD Biosciences	562438
Isotype control	PE	MOPC-21	Mouse IgG1	BD Biosciences	551436
Isotype control	PE	RMG2b-1	Mouse IgG2b	BioLegend	406708
Isotype control	BV711	MOPC-21	Mouse IgG1	BioLegend	400168
Isotype control	PE-Cy7	MOPC-21	Mouse IgG1	BioLegend	400126
Isotype control	APC-Cy7	MOPC-21	Mouse IgG1	BioLegend	400128
Isotype control	Alexa Fluor 700	MOPC-21	Mouse IgG1	BioLegend	400144
HLA-BC	PE	B1.23.2	Mouse IgG2b	eBioscience	12-5935-42
HLA-DR,DP,DQ	BV421	Tu39	Mouse IgG2a	BD Biosciences	564244
HLA-E	APC	3D12	Mouse IgG1	Miltenyi Biotec	130-120-071
Isotype control	PE	133303	Mouse IgG2b	R&D Systems	IC0041P
Isotype control	BV421	MOPC-173	Mouse IgG2a	BioLegend	400260
CD20	APC	2H7	Mouse IgG2b	BioLegend	302310
Isotype control	APC	MPC-11	Mouse IgG2b	BioLegend	400322
CD34	PE	563	Mouse IgG1	BD Biosciences	550619
CD43	BV510	1G10	Mouse IgG1	BD Biosciences	563377
CD45	APC	2D1	Mouse IgG1	BioLegend	368512

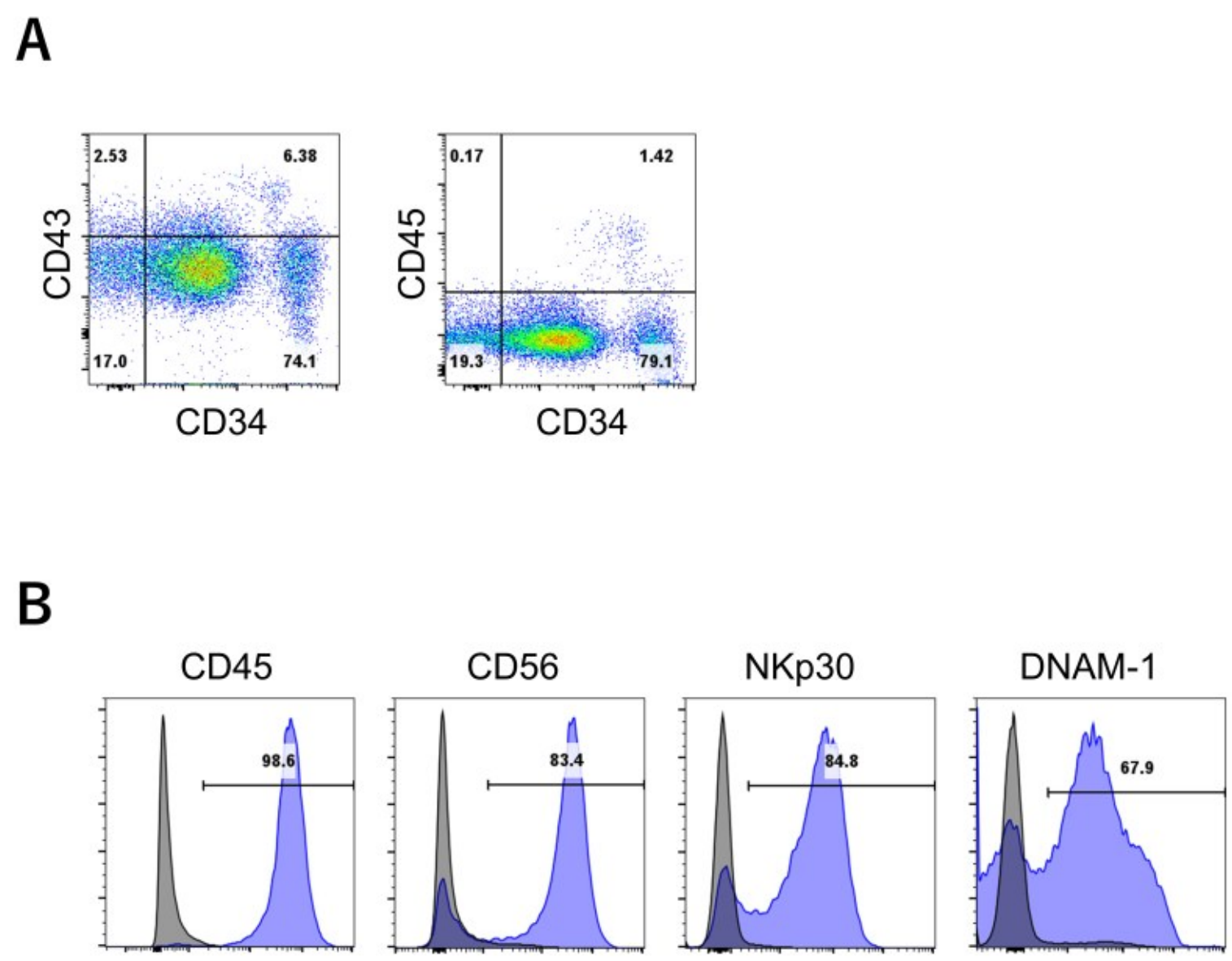

SUPPLEMENTARY FIGURE 1

In-process characterization of hematopoietic progenitor cell (HPC) and NK cell differentiation from UDC-iPSCs. **(A)** Flow cytometric analysis of cells harvested on day 13 of hematopoietic differentiation (Step 1). Representative plots show the emergence of CD34$^+$ hematopoietic progenitor cells and the appearance of CD34$^+$CD43$^+$ and CD34$^+$CD45$^+$ populations, consistent with progression toward definitive hematopoiesis. **(B)** Flow cytometric characterization of cells harvested on day 21 of NK cell differentiation (Step 2). Histograms show the expression of CD45, CD56, NKp30, and DNAM-1 on differentiating NK cells (blue) compared with isotype controls (gray). These data indicate successful progression from CD34$^+$ hematopoietic progenitors to CD45$^+$CD56$^+$ NK cells during the stepwise differentiation process.

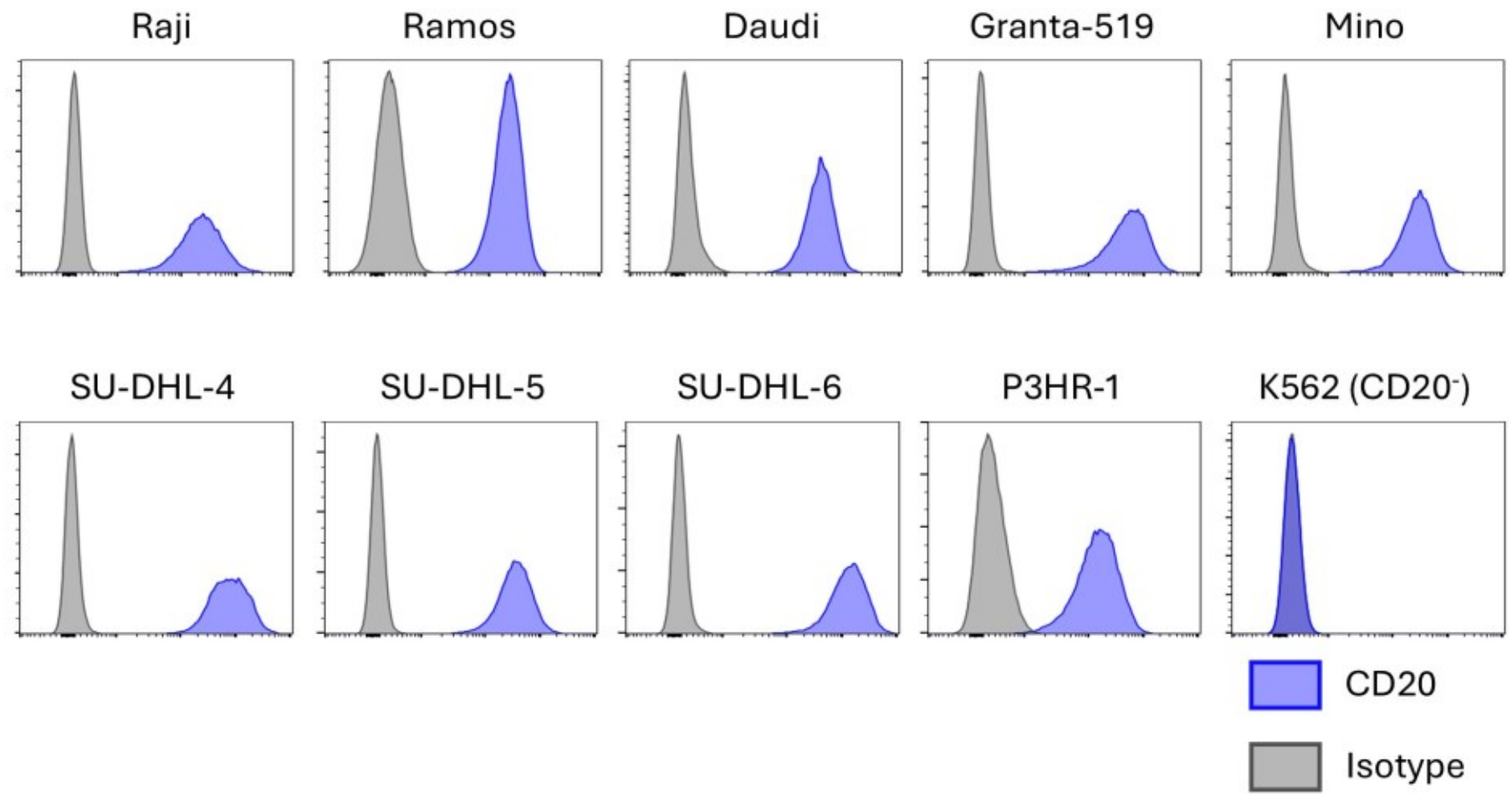

SUPPLEMENTARY FIGURE 2

CD20 expression across B lymphoma cell lines. The cells were stained with APC-labeled anti-human CD20 (blue) or APC-labeled isotype control antibody (grey) and analyzed by flow cytometry. See Supplementary Table S1 for antibody details.

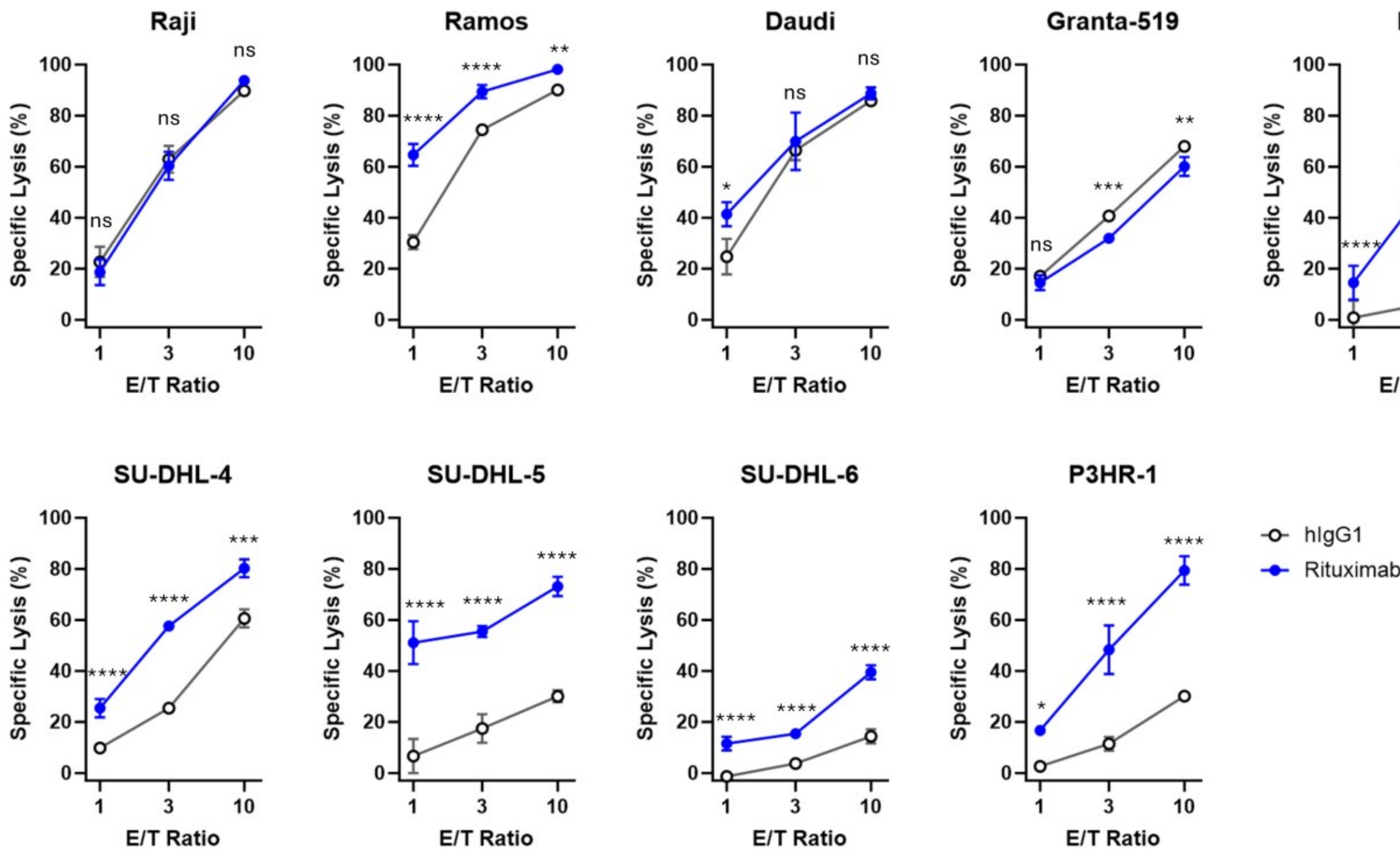

SUPPLEMENTARY FIGURE 3

Antibody-dependent cellular cytotoxicity (ADCC) of UDC-NK cells against a panel of B cell lymphoma cell lines. Target cells were labeled with CellTrace Violet and co-cultured with UDC-NK cells at an effector-to-target (E:T) ratio of 10:1 in the presence of 1 μg/mL Rituximab or a human IgG1 isotype control antibody for 4 hours. Following co-culture, cells were stained with viability dye and analyzed by flow cytometry. Target cell viability was determined by gating on CellTrace Violet–positive cells and assessing exclusion of the viability dye. Data are shown as mean ± SD from three independent experiments. ns, not significant, *P < 0.05, **P < 0.01, and P < 0.001, and *P < 0.0001.

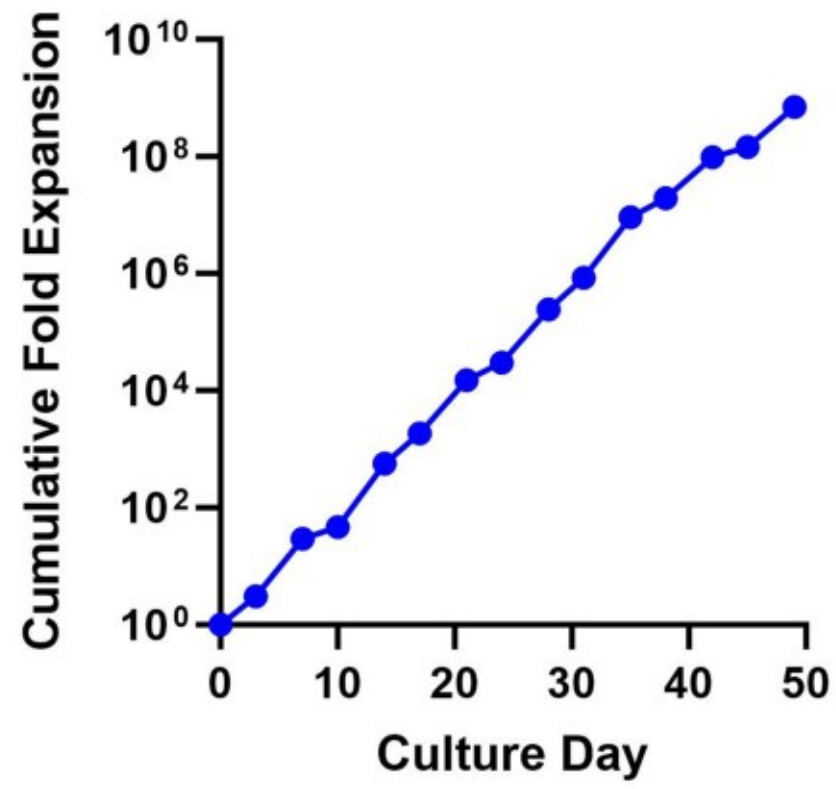

SUPPLEMENTARY FIGURE 4

Indefinite expansion of UDC-NK cells during an extended serial NK activation/expansion process. UDC-NK cells were expanded by repeating 3-day activation and 4-day expansion processes for 49 days.

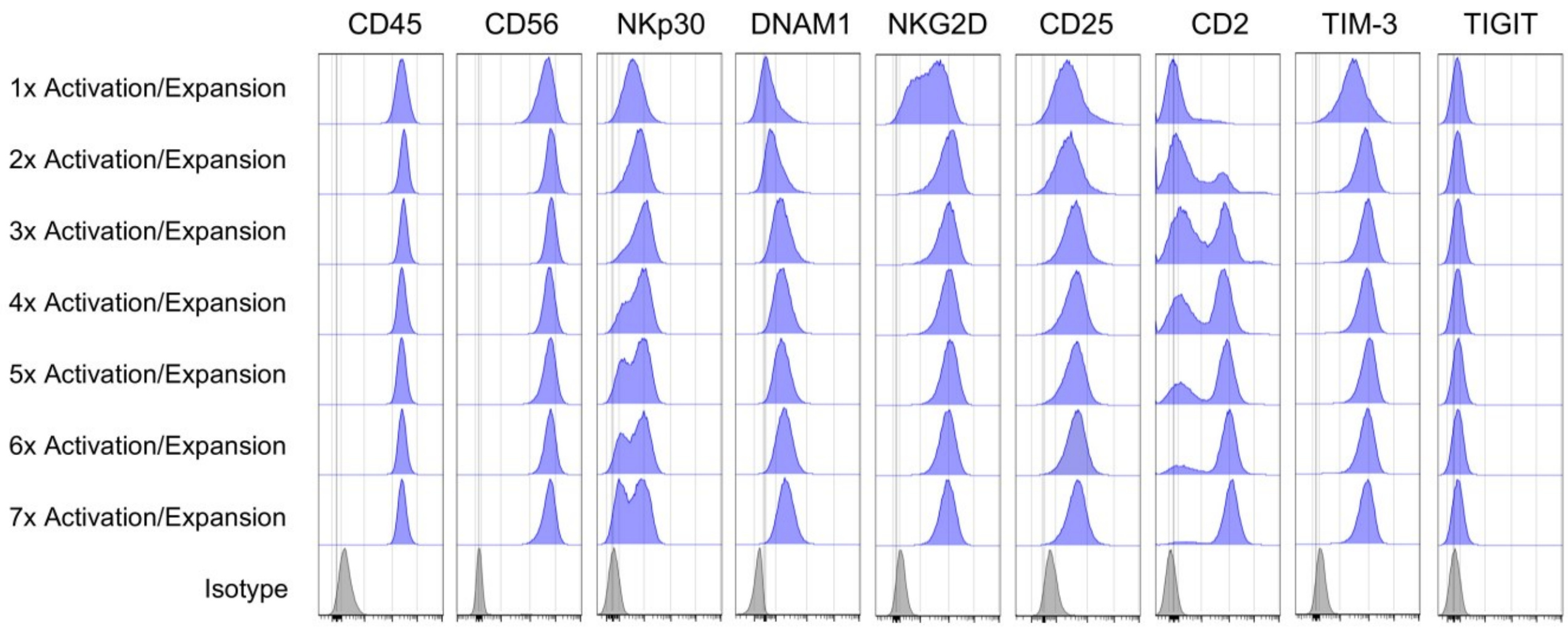

SUPPLEMENTARY FIGURE 5

Surface marker phenotype of cryopreserved UDC-NK cells during the serial NK activation/expansion process. Cryopreserved UDC-NK cells were thawed, immediately stained, and analyzed by flow cytometry. Canonical NK cell markers (blue) compared to isotype controls (grey). See Supplementary Table S1 for antibody details.

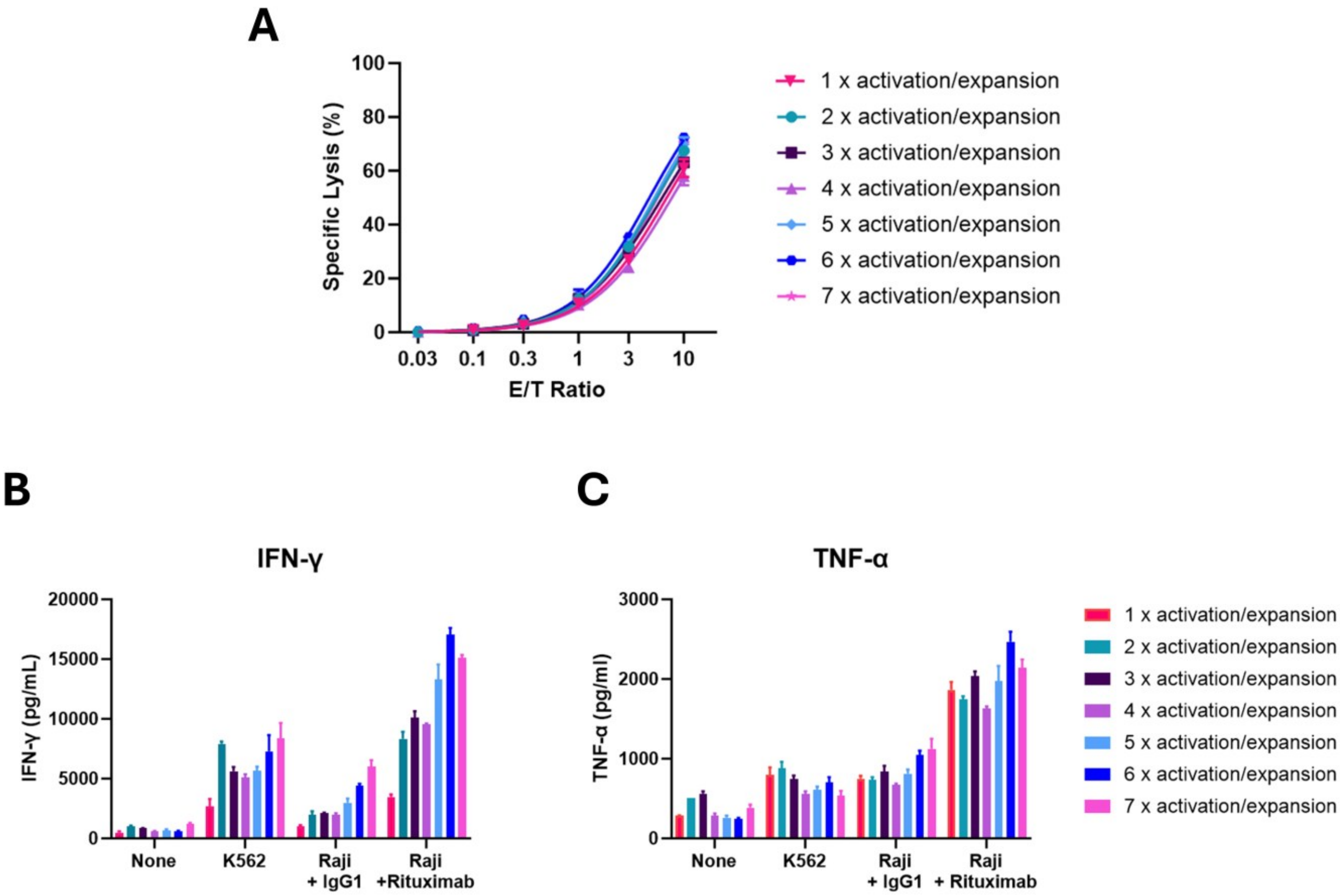

SUPPLEMENTARY FIGURE 6

In vitro function of UDC-NK cells during extended serial expansion. (A) UDC-NK cells harvested and cryopreserved during serial expansion were thawed and assessed for their cytotoxicity against K562 cells in a 4-hour cytotoxicity assay. The cytotoxicity was assessed by measuring HiBiT luciferase released from lysed K562 cells. Data are presented as mean ± SD from three independent experiments. (B) UDC-NK cells harvested and cryopreserved during serial expansion were thawed and co-cultured with K562 or Raji cells at a 1:1 E:T ratio in the presence or absence of 1 μg/mL Rituximab. After 24 hours of stimulation, culture supernatants were collected for cytokine analysis. Data are shown as mean ± SD from three independent experiments.

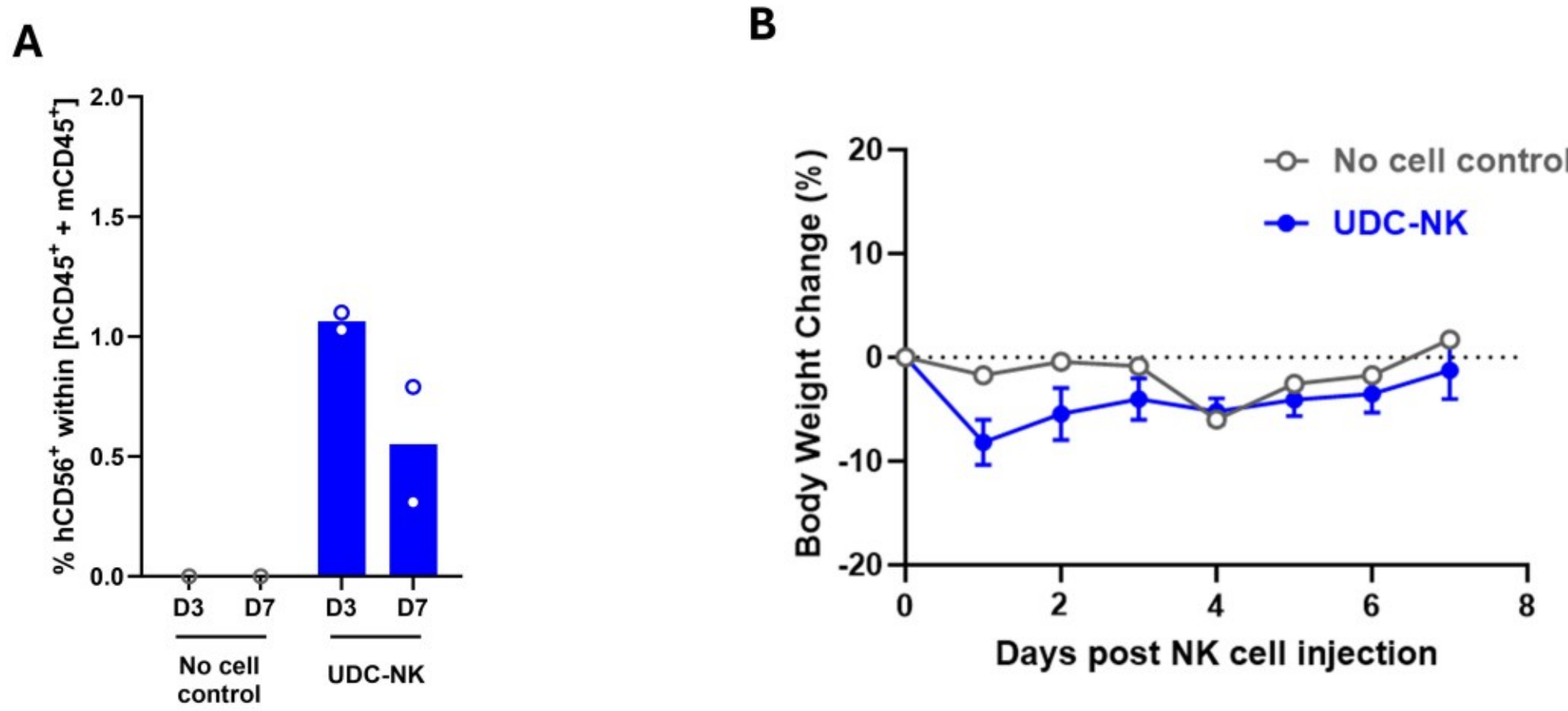

SUPPLEMENTARY FIGURE 7

In vivo persistence and tolerability of UDC-NK cells in mice. 1.5x107 cryopreserved UDC-NK cells were thawed and immediately injected intravenously into NOGF-IL-15 mice. Mice were carefully monitored to find any clinical signs, body weight loss, and macroscopic observations were for 7 days following NK cell injection. (A) To assess the persistence of UDC-NK cells, blood were harvested for flow cytometry. (No cell control n=1, UDC-NK n=2). (B) Body weight of mice after administration of UDC-NK cells. Data are presented as individual values, with the mean indicated for the UDC-NK group.
